# Supplementary figures and images for: Evolutionary dynamics of dengue virus in India
Source: PLoS Pathog. 2023 Apr 3;19(4):e1010862. doi: 10.1371/journal.ppat.1010862 (PMC10101646; doi:10.1371/journal.ppat.1010862)

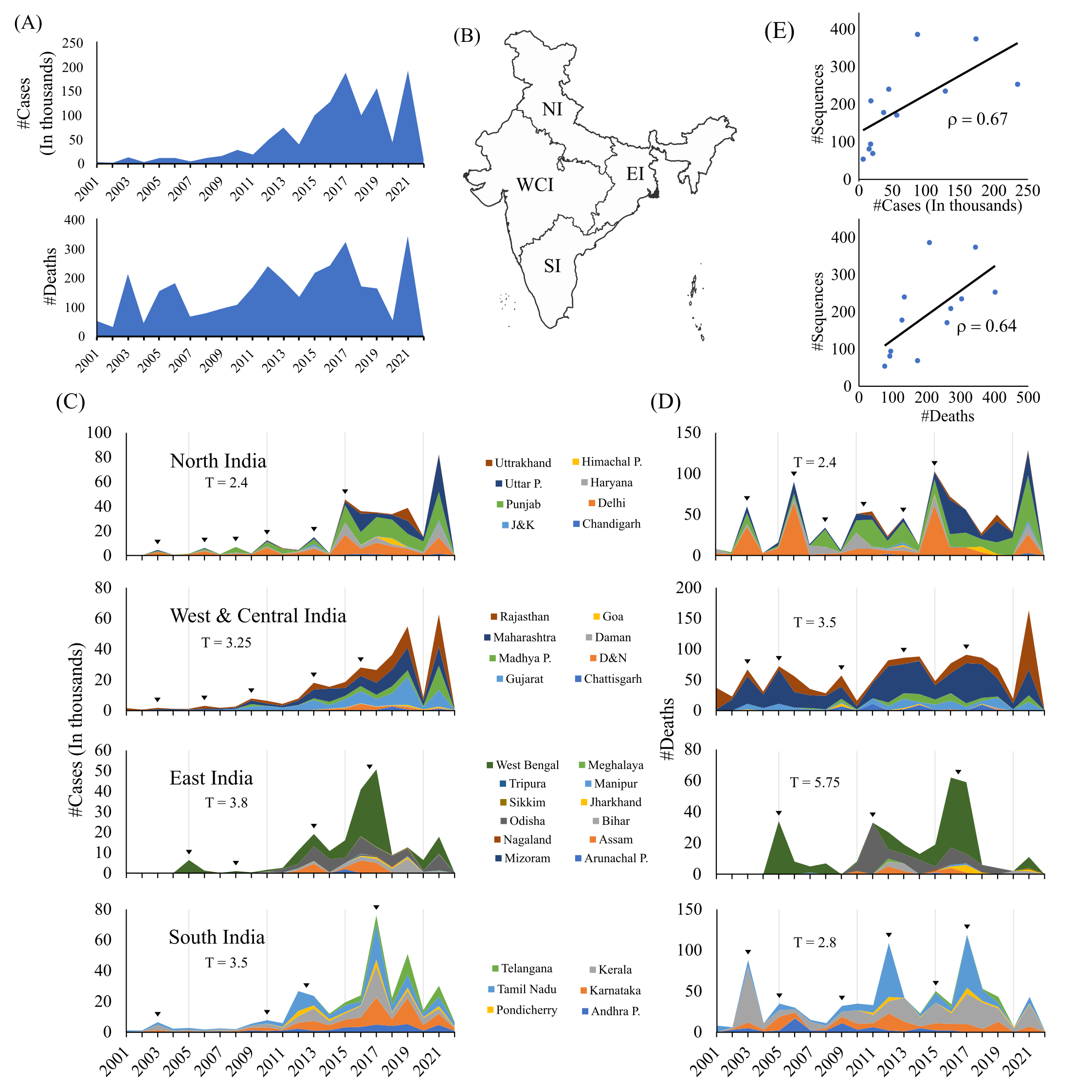

Supplement: S1 Fig — (A) Yearly dengue cases and deaths reported in India from 2001 to 2022 (B) Map of India denoting different regions (NI: North, EI: East, WCI: West & Central and SI: South India). The base map was obtained from https://mapsvg.com/maps/india, licensed under CC BY 4.0. The map was modified to show the region’s boundaries. (C) Yearly dengue cases and (D) deaths reported in different regions from 2001 to 2022. Data for states is colour coded in each plot. Black arrows denote peaks in the number of cases/death. The average time period between two consecutive peaks is reported for each case. Data from 2019–2022 was excluded while considering the peaks due to the effect of COVID-19-related disruptions. (E) Scatter plot showing the association between the number of dengue sequences from India and cases/ deaths reported. Pearson’s correlation coefficient is reported. (PNG) [file ppat.1010862.s001.png]

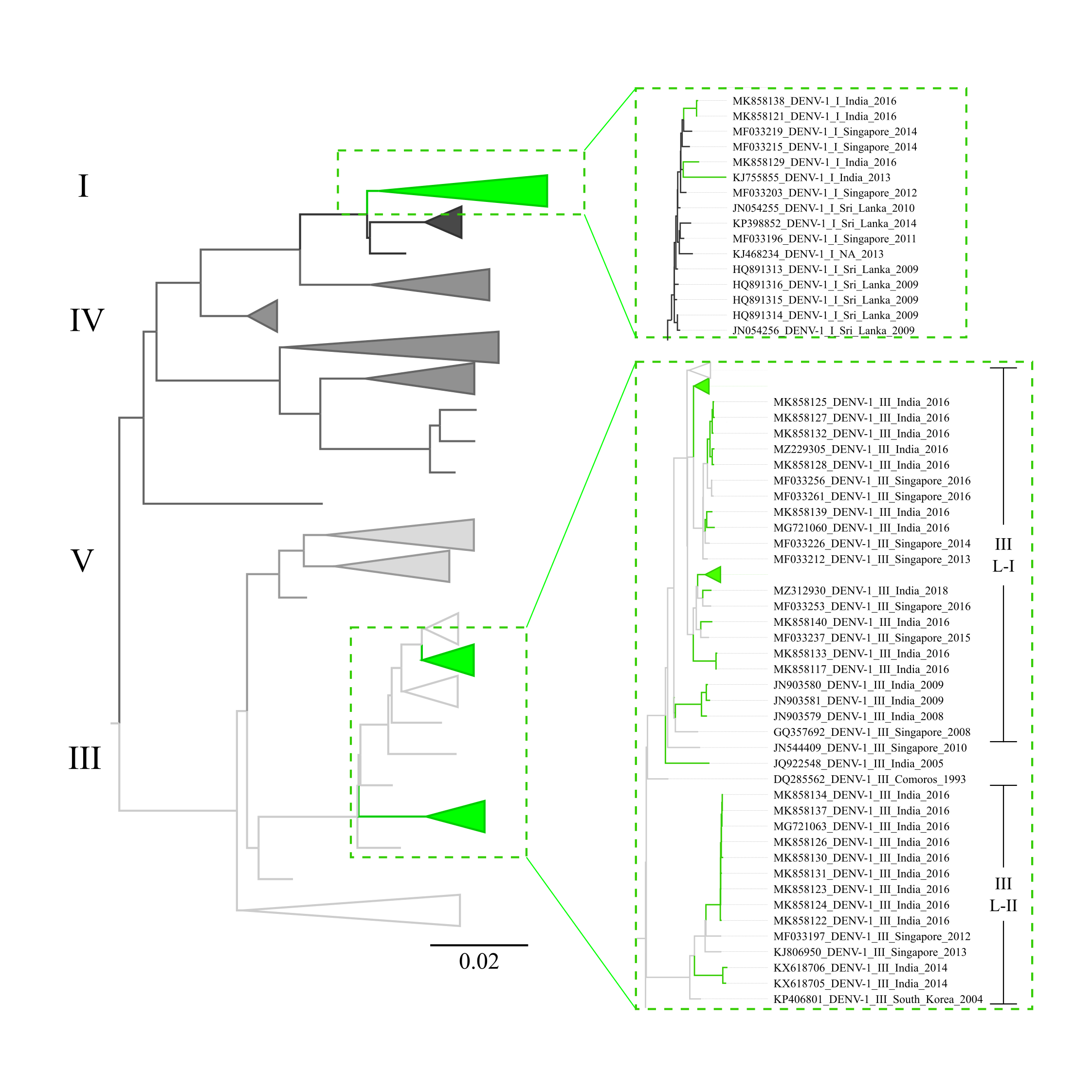

Supplement: S2 Fig — All available dated complete coding nucleotide sequences from the human infections were used for tree construction (n = 1800). Branches are collapsed to aid visualization. The detailed tree structure is shown for the neighbouring sequences in the insets. Grey shades within each tree show different genotypes, as indicated on the left side of each tree. The green regions carry the Indian sequences within the tree. The scale for the branch lengths is shown at the bottom of each tree. (PNG) [file ppat.1010862.s002.png]

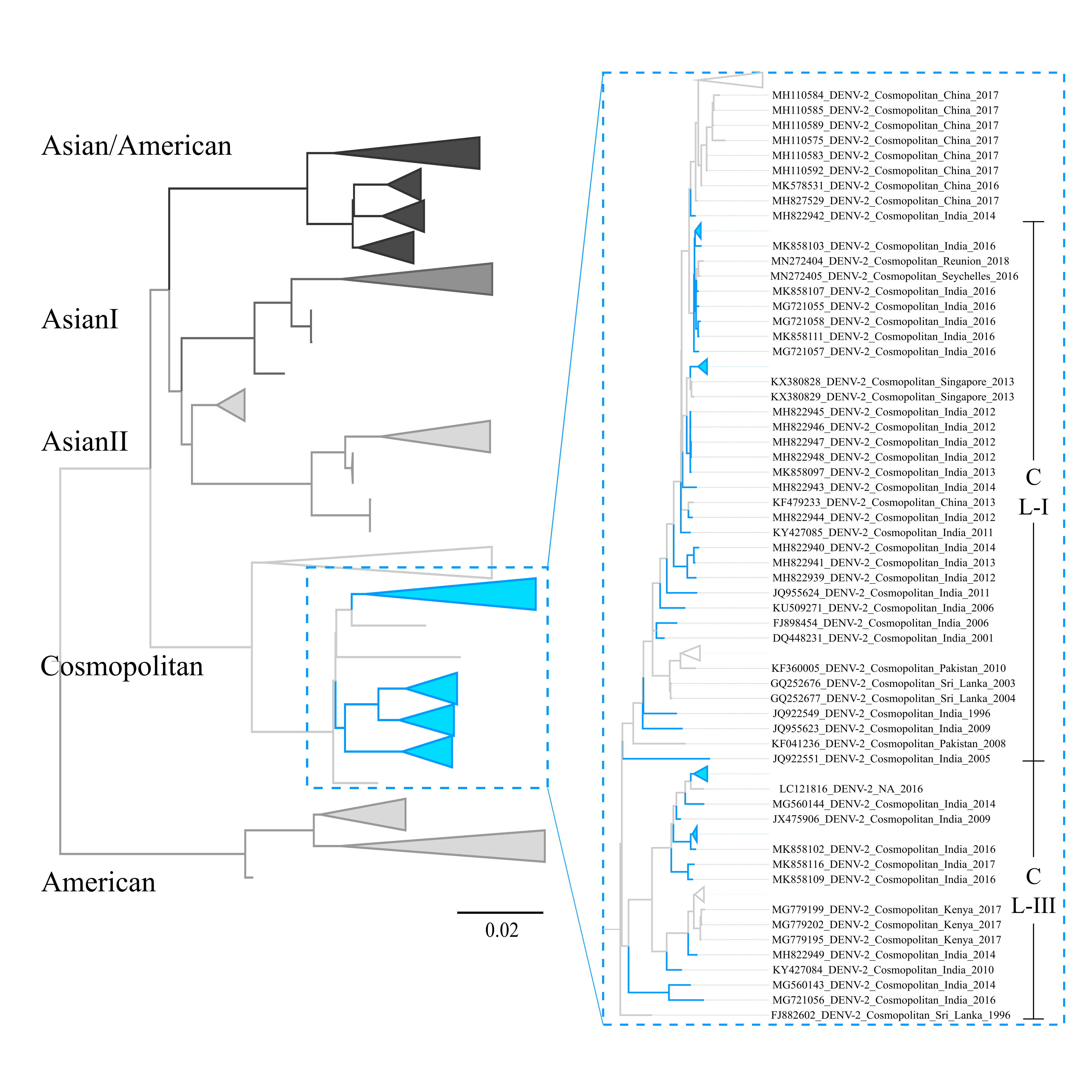

Supplement: S3 Fig — All available dated complete coding nucleotide sequences from the human host were used for tree construction (n = 1395). Branches are collapsed to aid visualization. The detailed tree structure is shown for the neighbouring sequences in the insets. Grey shades within each tree show different genotypes, as indicated on the left side of each tree. The blue regions show Indian sequences within the tree. The scale for the branch lengths is shown at the bottom of each tree. (PNG) [file ppat.1010862.s003.png]

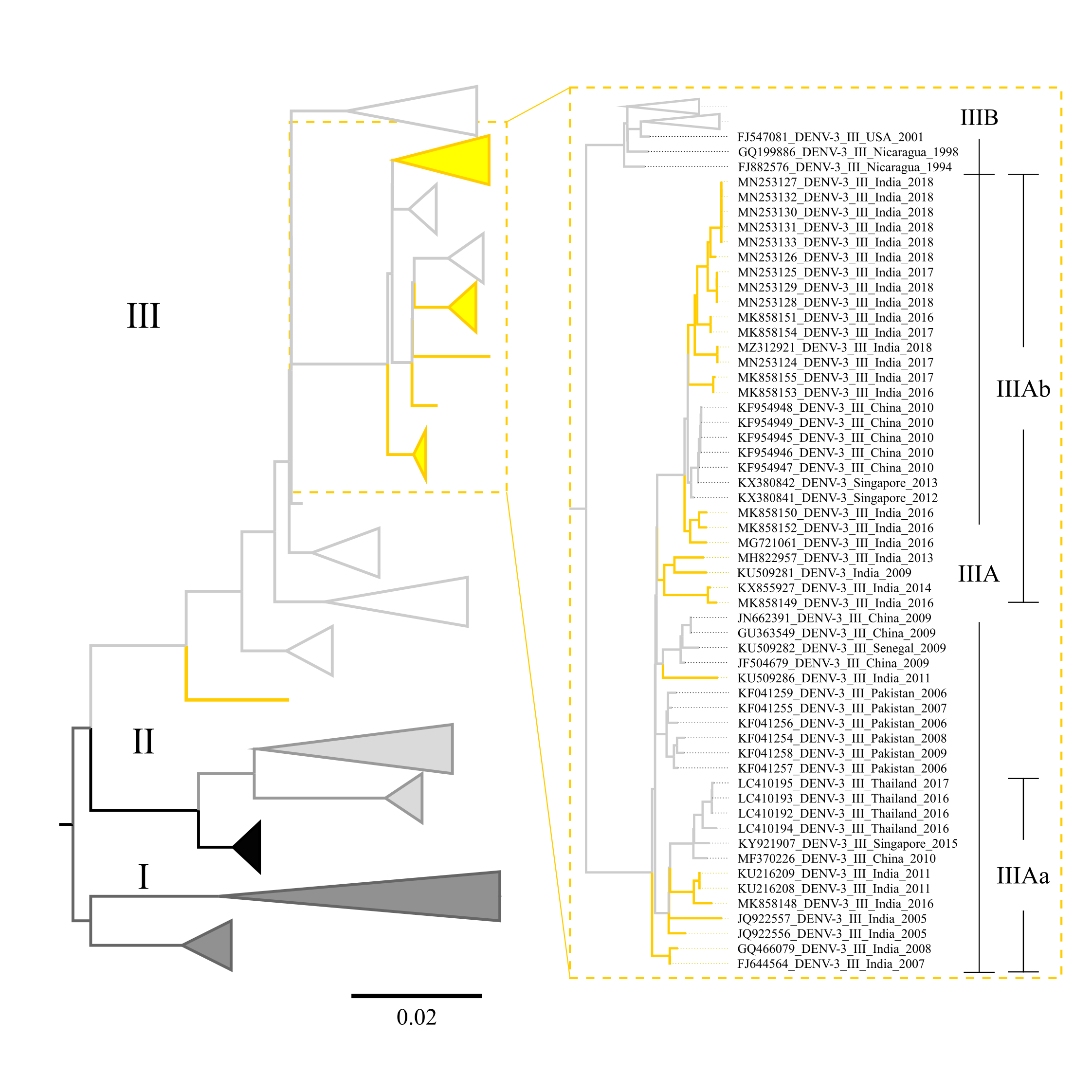

Supplement: S4 Fig — All available dated complete coding nucleotide sequences from the human host were used for tree construction (n = 823). Branches are collapsed to aid visualization. The detailed tree structure is shown for the neighbouring sequences in the insets. Grey shades within each tree show different genotypes, as indicated on the left side of each tree. The yellow regions highlight clades with Indian sequences within the tree. The scale for the branch lengths is shown at the bottom of each tree. (PNG) [file ppat.1010862.s004.png]

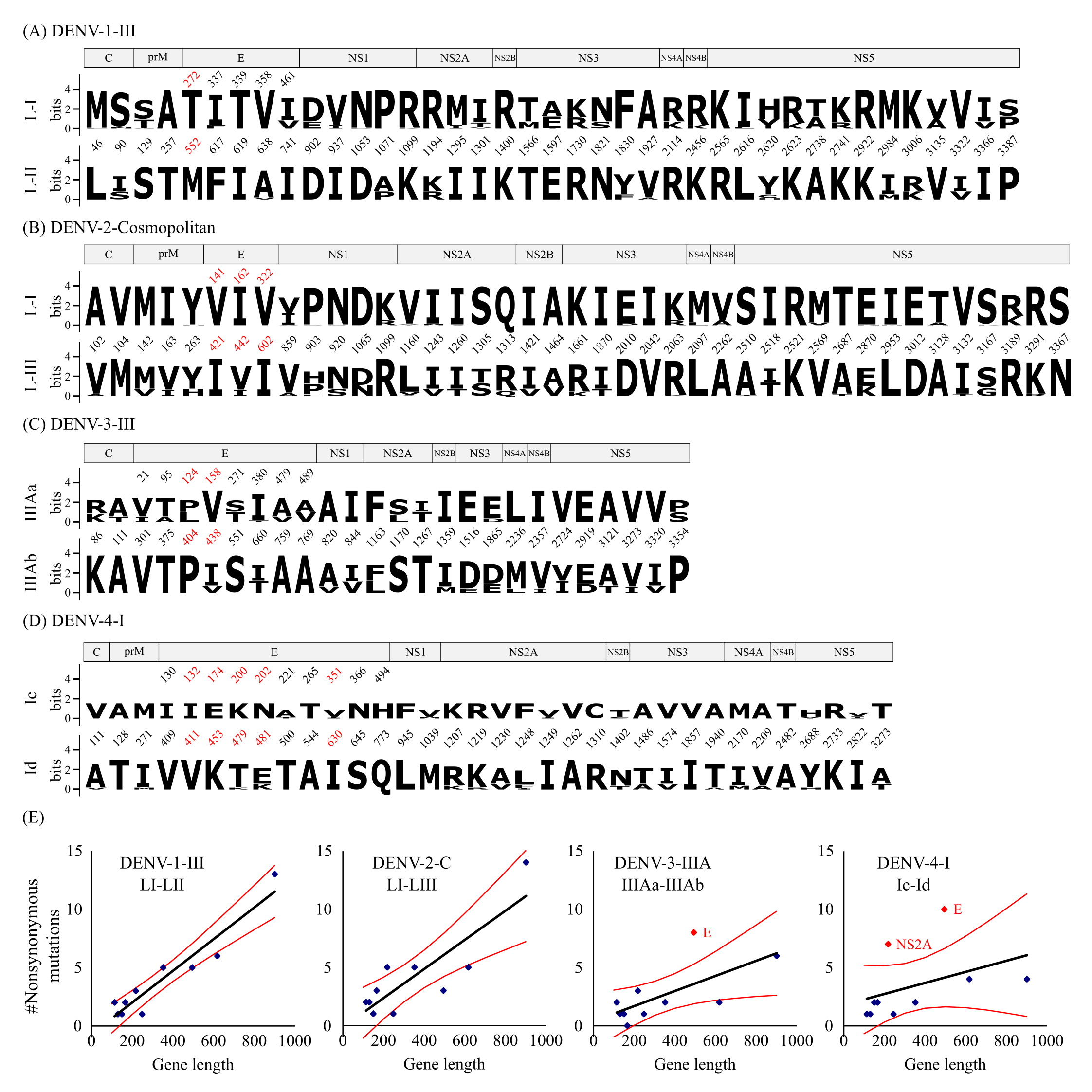

Supplement: S5 Fig — (A-D) Amino acid differences between the Indian lineages and sub-lineages are depicted using the sequence logo for each serotype with residue position in the dengue polyprotein listed in between. Gene boundaries are denoted on top, and amino acid positions within the E gene are shown below. The epitopic residue positions in the E gene are highlighted in red. (E) Association between the number of non-synonymous mutations and gene length for all the lineage/ sub-lineage pairs, red lines indicate 95% confidence interval for the regression line. The outlier points (in red) are denoted with the gene name. (PNG) [file ppat.1010862.s005.png]

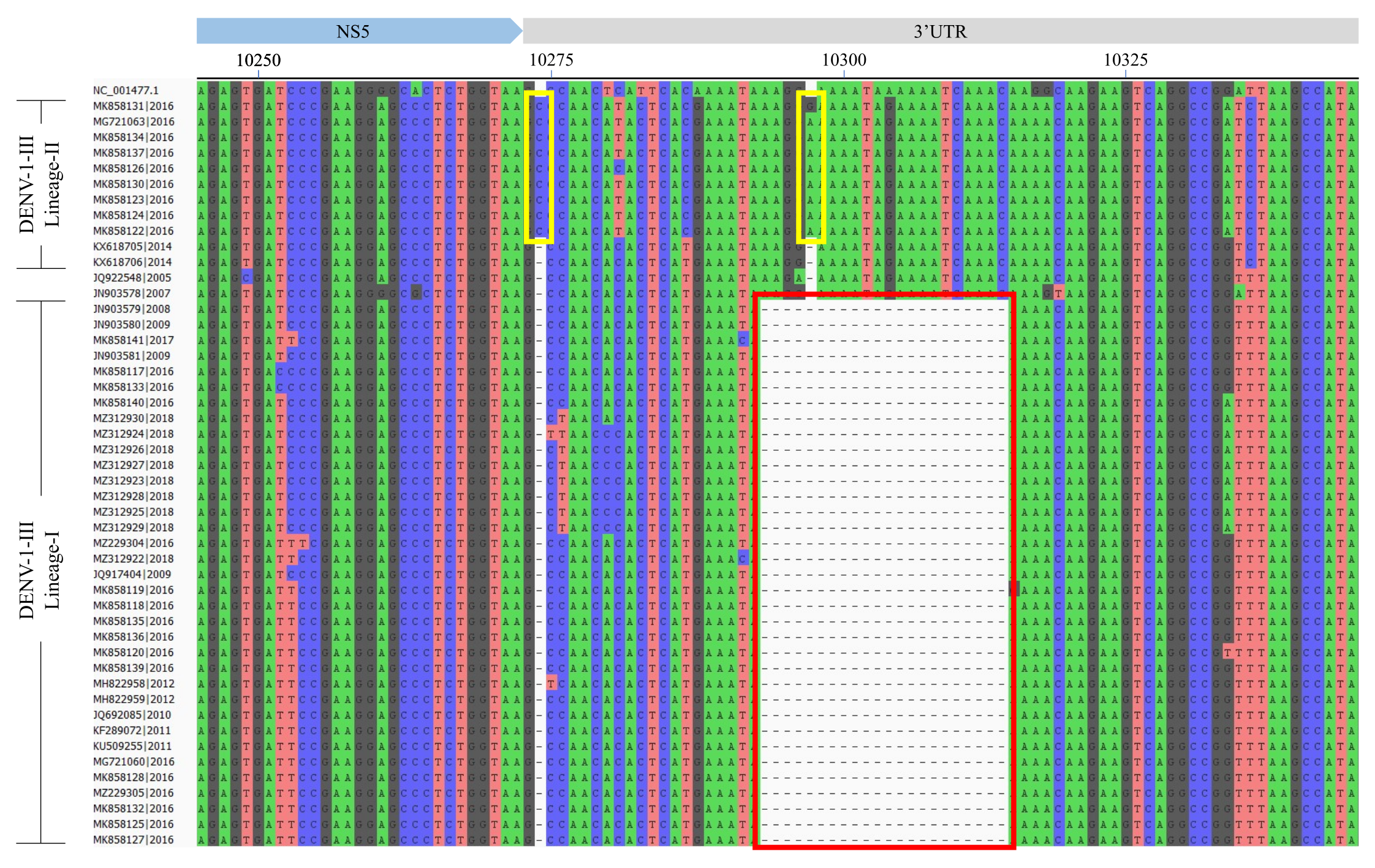

Supplement: S6 Fig — A deletion of 21 nucleotides in the variable region of 3’UTR of DENV-1-III lineage-I sequences is noted. Deleted nucleotide positions 10294–10314 with respect to the reference sequence are shown in the red box. Insertion of 2 nucleotides: C and A, at 10274 and 10297 in DENV-1-III lineage-II sequences, is shown in the yellow boxes. (PNG) [file ppat.1010862.s006.png]

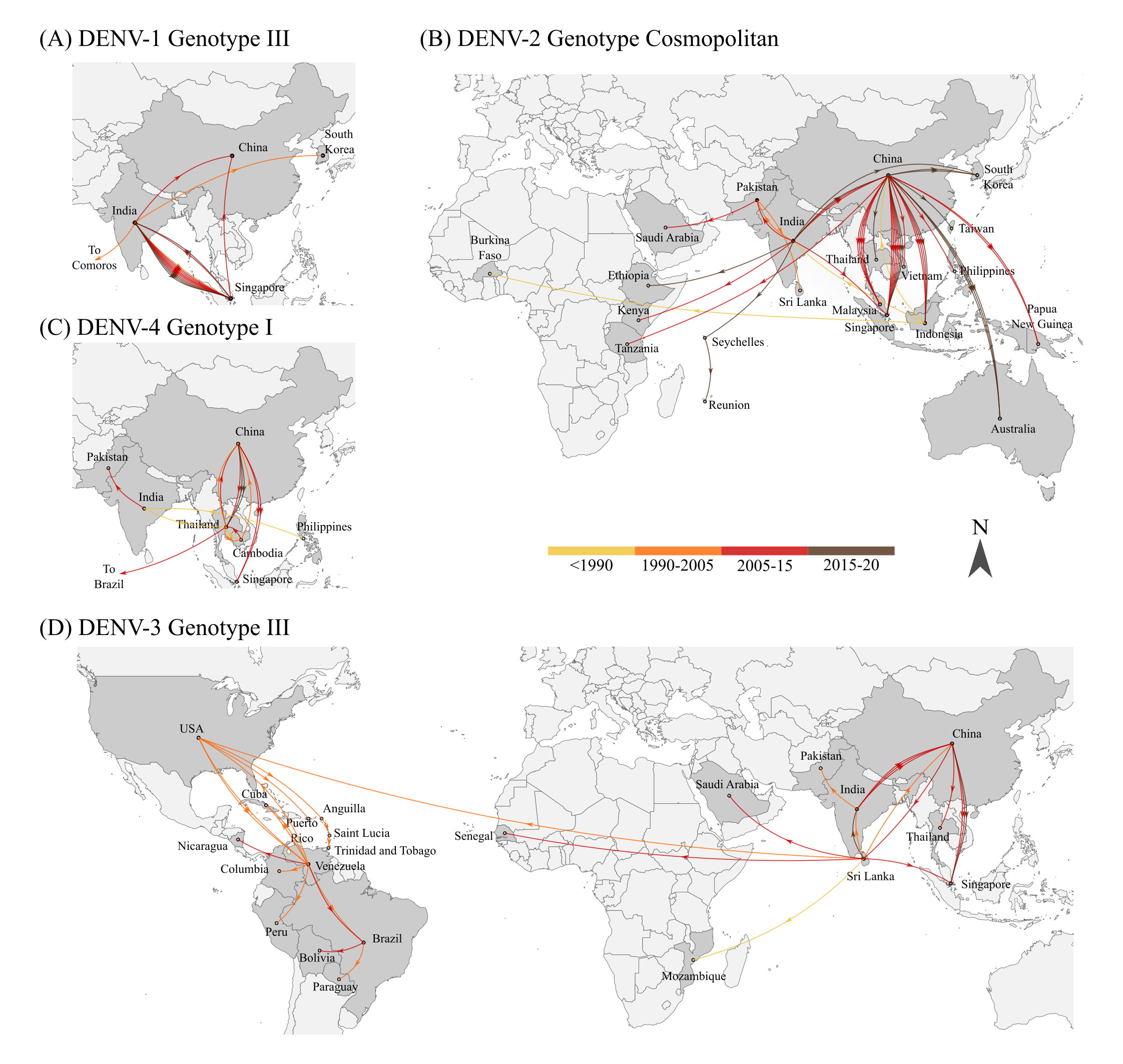

Supplement: S7 Fig — The movement of the dengue virus within different countries is shown for all serotypes. (A-D) Countries involved in the spread are shown in dark grey. Arrow lines represent the probable direction of the spread. The predicted time of virus introduction to the country is colour coded and shown at the bottom. The base map of the world was obtained from https://mapsvg.com/maps/world, licensed under CC BY 4.0. (PNG) [file ppat.1010862.s007.png]

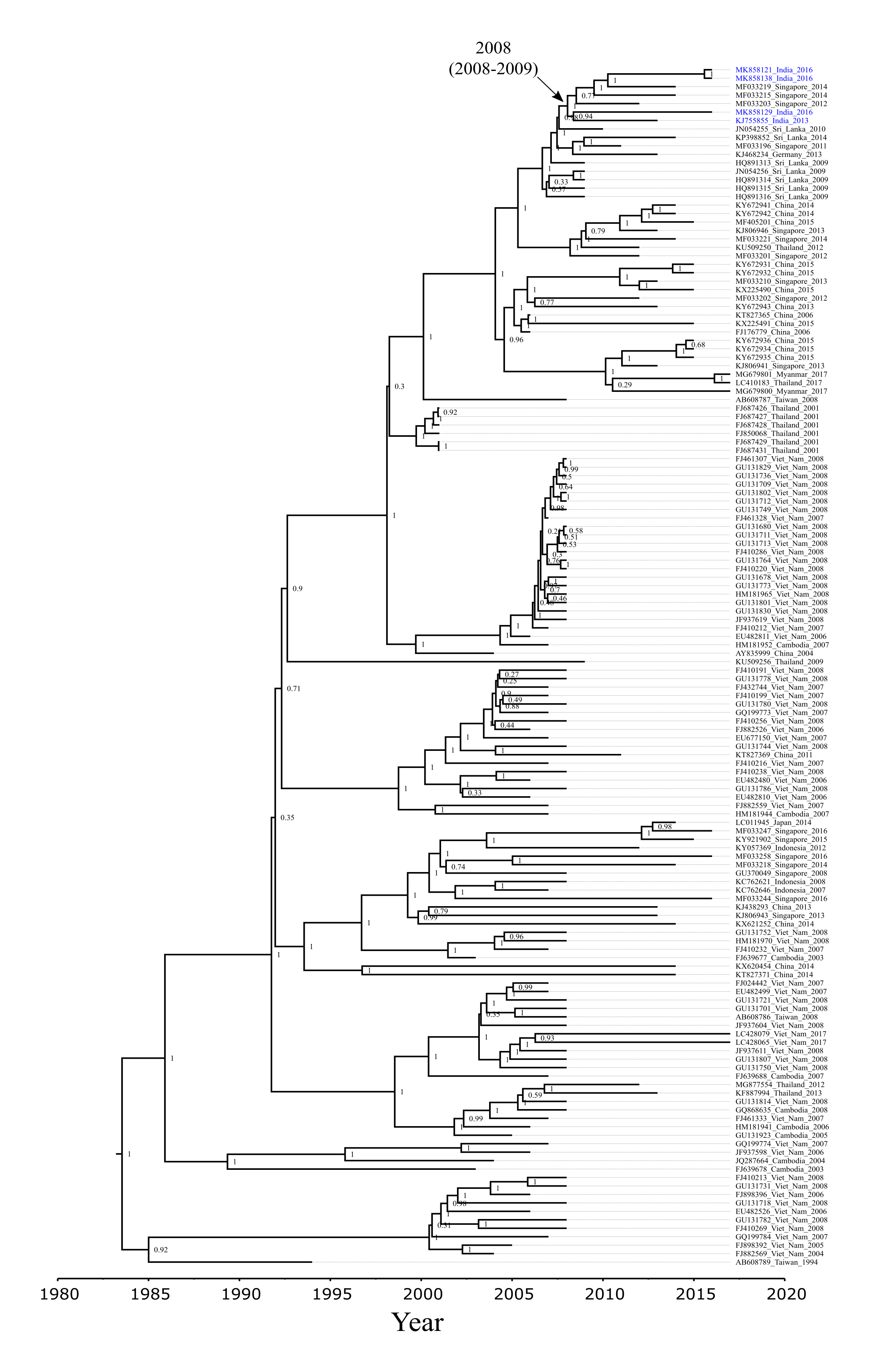

Supplement: S8 Fig — Time-dated trees for DENV-1-I. The x-axis denotes the time in years. The time of the year at important nodes is shown along with the 95% HPD. The posterior probability is shown at each node. (PNG) [file ppat.1010862.s008.png]

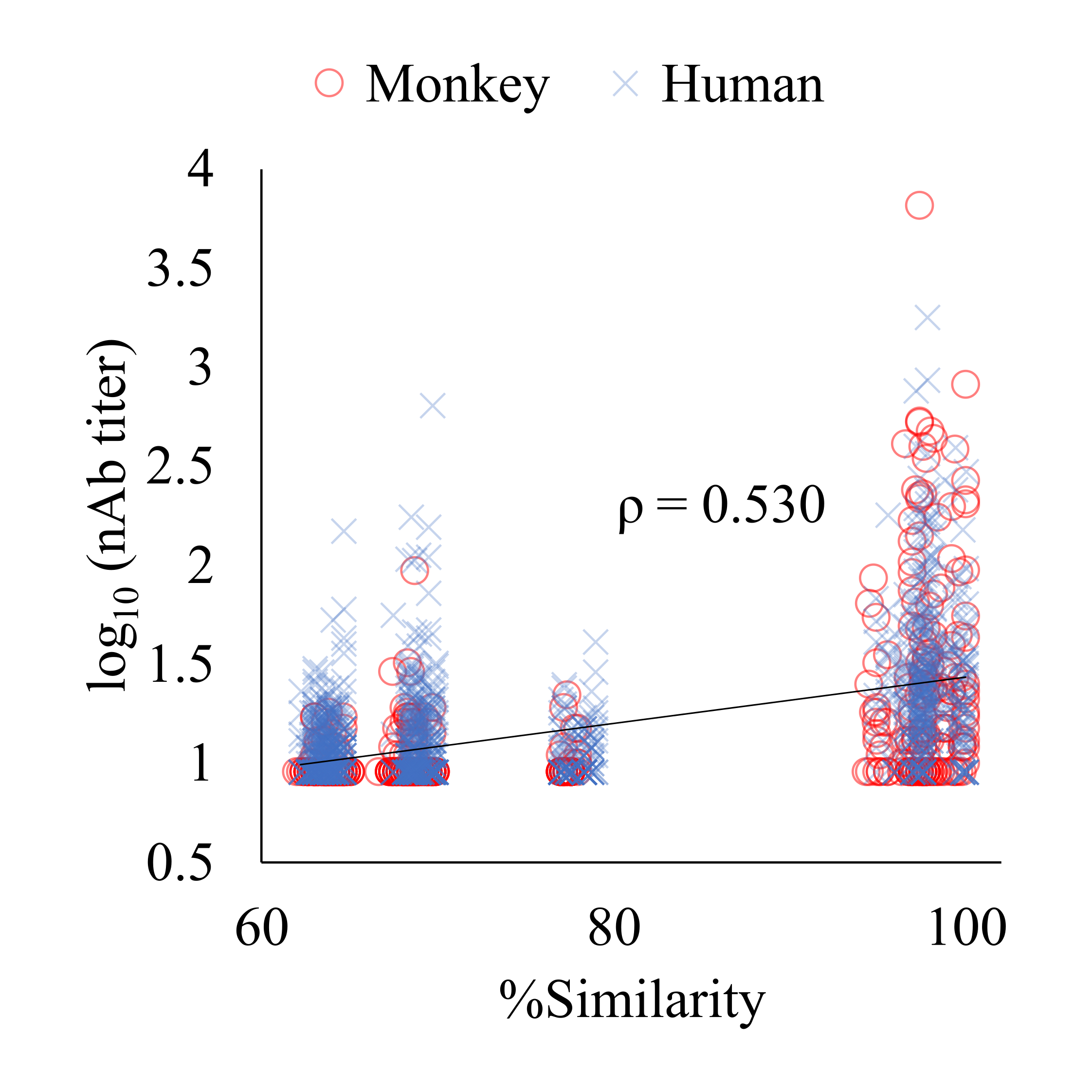

Supplement: S9 Fig — Correlation between neutralization antibody titer collected from humans (x) collected post-vaccination and African green monkeys (o) collected post-infection and tested against a panel of viruses. The X-axis denotes the percentage similarity between the amino acid sequences of the infecting and test viruses. (PNG) [file ppat.1010862.s009.png]

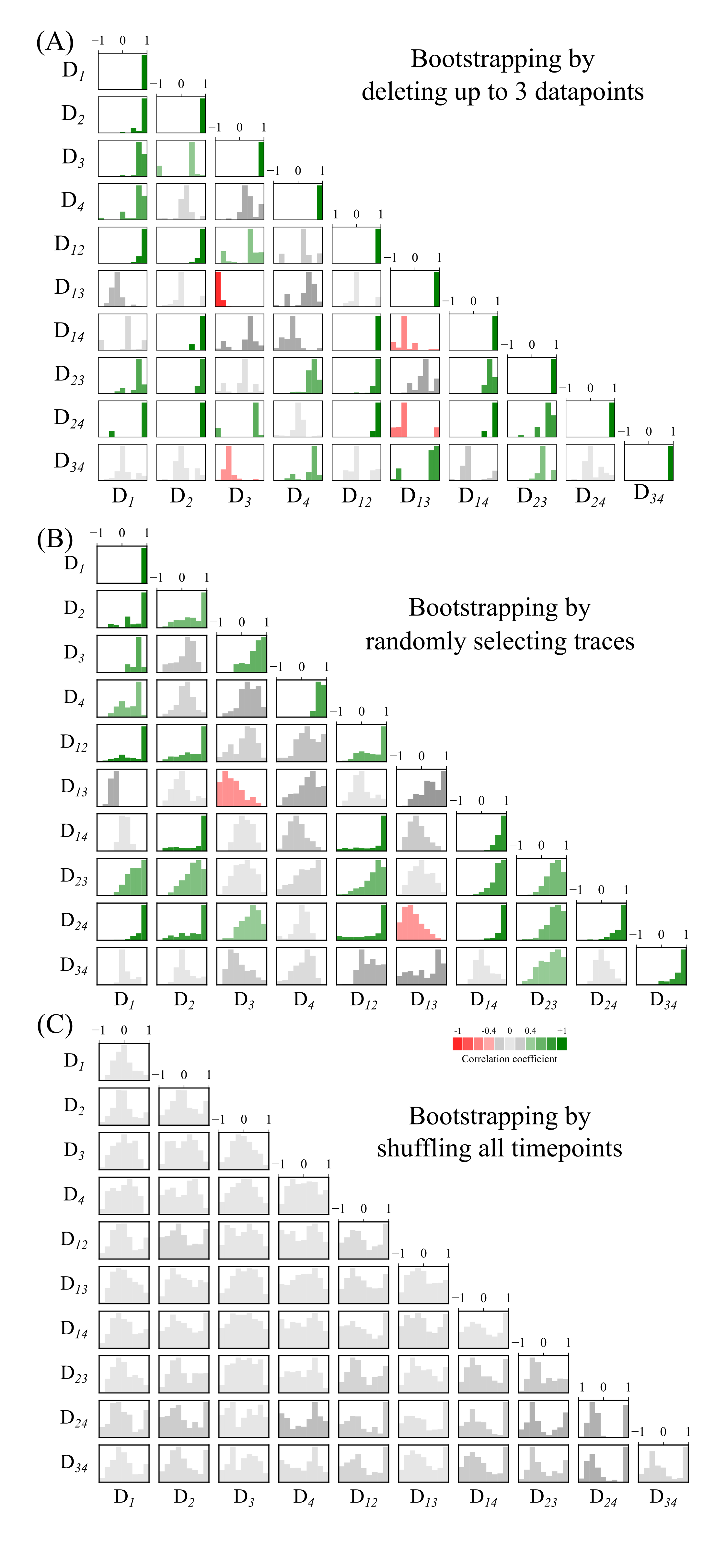

Supplement: S10 Fig — (A) Distributions were obtained by deleting up to 3 data points randomly from individual dynamics (1000 bootstraps). (B) Histograms of correlation coefficients were obtained by randomly selecting the individual traces (1000 bootstraps). (C) Histogram of correlation coefficients obtained by shuffling the time points randomly (1000 bootstraps). Green, red and grey indicate positive (ρ>0.4), negative (ρ<-0.4), and weak correlation. (PNG) [file ppat.1010862.s010.png]

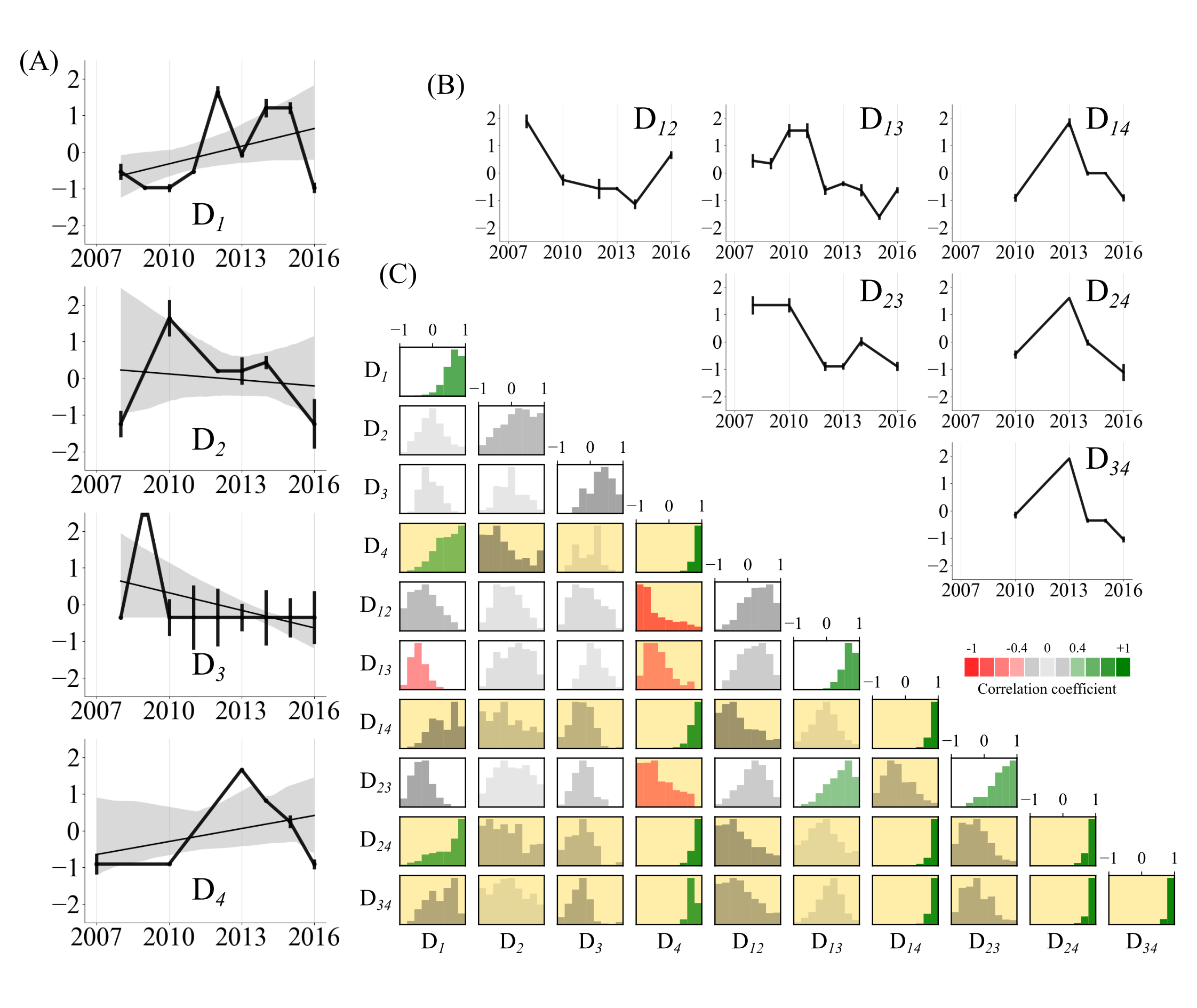

Supplement: S11 Fig — (A) Normalized amino acid distances with respect to the 2007/2008 dengue strains is plotted in black. Hamming distances were normalized with the sequence length and converted to a z-score. Distances were bootstrap sampled (n = 100) to calculate the reported median. Error bars represent standard error. A line and grey shaded region represent a linear regression line with an 80% confidence interval. Di indicates the distance dynamics between DENV-i from its (2007/2008) ancestral sequence. (B) Year-wise normalised interserotype distances (as z-score) are reported as calculated by randomly selecting the sequences from each serotype every year (100 bootstraps) is plotted. Dij indicates the interserotype distance dynamics between i-th and j-th serotype. (C) Distribution of correlation coefficients for within-serotype dynamics and inter-serotype distance dynamics. Histograms were obtained by deleting up to 3 data points randomly (1000 bootstraps). Correlations involving the DENV-4 serotype are highlighted with yellow background and ignored due to the lack of enough sequences. Green and red indicate positive (ρ>0.4) and negative correlation (ρ<-0.4), respectively, while grey indicates weak or no correlation. (PNG) [file ppat.1010862.s011.png]

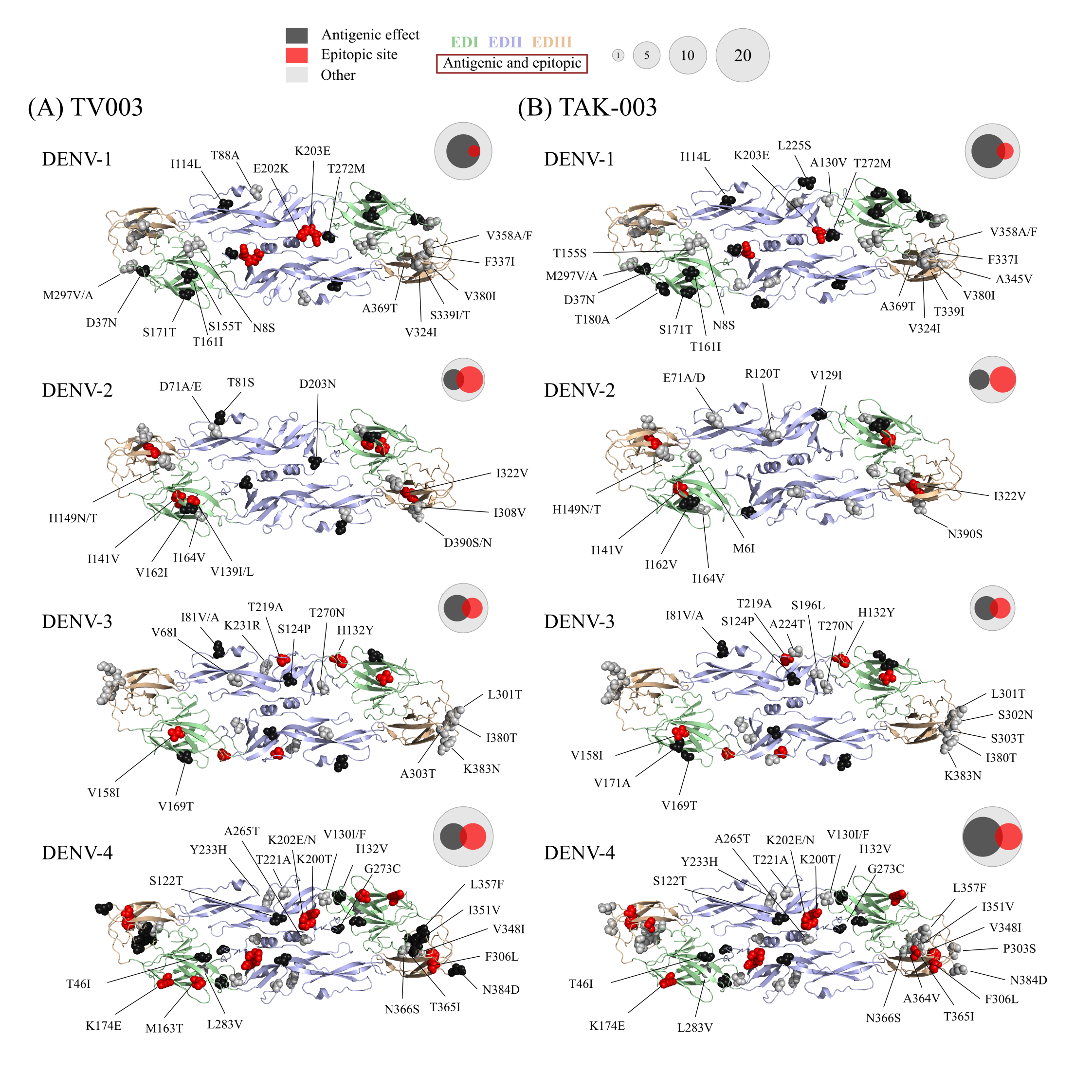

Supplement: S12 Fig — Amino acid differences at a frequency >10% with respect to (A) TV003 and (B) TAK-003 vaccine are shown on the envelope protein dimer structure in grey, red and black. Different positions in the known epitopic regions are shown in red. Positions in the predicted antigenic effect positions are shown in black. Residues present in epitopic regions with predicted antigenic effects are highlighted with a box. Venn diagram shows the total number of differences in the full E protein (including the stem and transmembrane region), epitopic sites and antigenic sites. The circle size represents the number of differences category-wise. (PNG) [file ppat.1010862.s012.png]
