# Supplementary material for: Evolutionary dynamics of dengue virus in India
Source: PLoS Pathog. 2023 Apr 3;19(4):e1010862. doi: 10.1371/journal.ppat.1010862 (PMC10101646; doi:10.1371/journal.ppat.1010862)
Supplement: Attachment — Submitted filename: Response to reviewers.pdf [file ppat.1010862.s021.pdf]

Dear Dr. Roy,

Thank you very much for submitting your manuscript "Evolutionary dynamics of dengue virus in India" for consideration at PLOS Pathogens. As with all papers reviewed by the journal, your manuscript was reviewed by members of the editorial board and by several independent reviewers. In light of the reviews (below this email), we would like to invite the resubmission of a significantly-revised version that takes into account the reviewers' comments.

Most of the reviewers indicate that this study, a detailed evaluation of dengue virus genetic diversity in India, addresses an important gap in the literature. However, all reviewers were concerned about over-interpretation of results, especially given limitations in antigenic data and other epidemiological data. Addressing the reviewers' comments about areas of the manuscript that are unclear, toning down interpretations beyond what is supported by the data, clearly stating limitations, and including additional methodological details and phylogenetic analyses as suggested should be addressed prior to publication. Reviewer 2 also proposed including clinical and epidemiological data to support the genetic analyses. We understand inclusion of such datasets may not be possible. If the other reviewer comments and concerns about the text and additional analyses can be addressed, this would make the manuscript suitable for publication.

We cannot make any decision about publication until we have seen the revised manuscript and your response to the reviewers' comments. Your revised manuscript is also likely to be sent to reviewers for further evaluation.

When you are ready to resubmit, please upload the following:

[1] A letter containing a detailed list of your responses to the review comments and a description of the changes you have made in the manuscript. Please note while forming your response, if your article is accepted, you may have the opportunity to make the peer review history publicly available. The record will include editor decision letters (with reviews) and your responses to reviewer comments. If eligible, we will contact you to opt in or out.

[2] Two versions of the revised manuscript: one with either highlights or tracked changes denoting where the text has been changed; the other a clean version (uploaded as the manuscript file).

Important additional instructions are given below your reviewer comments.

Please prepare and submit your revised manuscript within 60 days. If you anticipate any delay, please let us know the expected resubmission date by replying to this email. Please note that revised manuscripts received after the 60-day due date may require evaluation and peer review similar to newly submitted manuscripts.

Thank you again for your submission. We hope that our editorial process has been constructive so far, and we welcome your feedback at any time. Please don't hesitate to contact us if you have any questions or comments.

Sincerely,

Leah Katzelnick

Guest Editor

PLOS Pathogens

Sonja Best

Section Editor

PLOS Pathogens

Kasturi Haldar

Editor-in-Chief

PLOS Pathogens

orcid.org/0000-0001-5065-158X

Michael Malim

Editor-in-Chief

PLOS Pathogens

orcid.org/0000-0002-7699-2064

****

We thank the reviewer for the comments and raising some important points. We have addressed all the queries, revised the manuscript significantly, added necessary details and made it easier to read and understand. Please find our point-by-point response and revisions implemented below.

Reviewer's Responses to Questions

Part I - Summary

Please use this section to discuss strengths/weaknesses of study, novelty/significance, general execution and scholarship.

Reviewer #1: This is an important study considering the genetic diversity of dengue in India, an area about which there is little known for dengue. I found some of the manuscript a little confusing, and required some further clarifications on the methods and on the reasoning for some of the conclusions drawn.

We agree with the reviewer that for a nation of 1.3 billion people, current knowledge of an endemic disease like Dengue is sporadic and insufficient. Our hope is to alleviate this with coordinated and dedicated efforts. This is the first manuscript focusing on Dengue diversity across India laying the foundations for future and more detailed studies.

We are sorry to hear that the reviewer found certain segments of the manuscript difficult to understand. We have added clarifications and elaborated our reasoning wherever suggested.

Reviewer #2: The manuscript describes the evolutionary dynamics of dengue virus in India. Overall, the manuscript sufficiently reports finding and provides thorough discussion. However, there are some limitations regarding the general findings, in which no specific or breakthrough findings were described. The cyclical patterns of dengue virus serotype is a common phenomenon in all endemic countries. The genotypes co-circulation and replacement is also commonly observed in other countries.

We agree with the reviewer that cyclical pattern, co-circulation and replacement of serotypes have been reported before in some countries and anecdotal evidence does suggest cyclical pattern in India. However, as per our knowledge, this is the first time the serotype distribution dynamics is reported from such analysis across India.

The significance of our manuscript lies beyond that. Due to a substantially larger dataset and careful comparison of temporal evolution across all dengue genomes sequenced till date, we were able to identify previously unreported cyclical patterns in dengue virus evolution. Due to the strong fluctuations observed in the antigenic Envelope (E) gene (Fig 4) compared to CprM gene (see S11 Fig in the manuscript), we argue that such evolutionary fluctuations are driven by immune selection. Moreover, the comparable time scale between the dengue peak incidences and fluctuations in the E gene evolutionary dynamics point to the intricate relationship between the E gene evolution and the epidemic dynamics. Of course, it warrants further research to see

if there is a direct causal relationship between the two. However, such linkage between dengue virus evolution and population immunity in India has never been suggested by others before. If true, it could explain similar cyclical patterns observed in other epidemic countries as well.

Further, we identify a new variant of DENV4 (Id lineage) that is currently propagating in India. Compared to its closest lineage Ic, the mutations in the E gene are focused in antigenic sites and the virus has acquired amino acids akin to residues in DENV1 and DENV3 in India. It is unlikely to be a coincidence and suggests that the E gene in DENV4-Id lineage is evolving under the influence of interserotypic effects.

On similar lines, DENV E gene in India strains is highly divergent compared to current dengue vaccine strains and majority of the differences again cluster on the antigenic sites in the E gene. This raises the possibility of substantial reduction in efficacy of these vaccines against Indian strains as has been shown for similar mutations (as discussed in detail in the manuscript).

Overall, our work suggests for the first time that immune selection is a key driver for dengue virus evolution in India and showcases some of its consequences. Several of our findings has important implications for dengue vaccine trials and public health in India.

Moreover, although the methods and analyses were sufficient, the sample number is probably not strong enough to be representative of the whole India, especially because there are 4 DENV serotypes present that need to be equally represented. Authors may try to explain the sample size in term of their representative-ness in the manuscript, both serotypically and geographically. Given the massive population of India, the authors must be able to convince the reader that the sample size is powerful enough to draw conclusion.

We understand the reviewer's concern regarding the sample size and representativeness. We have addressed this issue in the part-II of the response to reviewer's comment (see below).

The manuscript may be more useful if it includes the clinical and epidemiological data so that the viral genetic data will be more meaningful and may be used to mitigate future epidemics. This, and other limitations, are not specifically described in the manuscript.

We agree with the reviewer that the clinical and epidemiological data would strengthen our observations and provide new insights that are even beyond the current manuscript. In the current study, we have tried to comprehensively cover all available genomic data from the Genbank and/or ViPR database. Unfortunately, clinical metadata is not available corresponding to many of the Indian virus sequences. This limits our ability to draw causal inferences only regarding genetic evolution of the virus in India with broad generalizations.

Mitigation of future epidemics is a key motivation of our work. However, prediction of future epidemics requires coordinated efforts to incorporate studies like ours in better multi-parametric models. Such models must incorporate and analyze data on factors that influence dengue transmission, population immunity, virus evolution, climate, population density, and urbanization among others. This is unfortunately beyond the scope of our current manuscript but will be part of future work.

Inspite of these limitations, our manuscript discusses several epidemiological insights including lineage/sublineage changes, co-incidence of outbreaks with serotype replacement events, emergence of new strains, co-circulation of closely related genotypes and the dynamics of genetic changes in the Indian dengue strains in the results section.

DENV1 genotype III, the dominant genotype in India has two lineages (lineage-I and II) co-circulating in the country (Results: Line 253-258). While DENV1-III lineage-I is present across the country, lineage-II is circulating only in South India (Results: Line 266-269). A distinct DENV1-I genotype was introduced in South India briefly between 2012-15 but not reported since. For DENV2, the cosmopolitan lineage I and III are reported extensively from across the country. Our group has previously shown that the replacement of lineage-I by lineage III in New Delhi even though they don't vary in terms of growth kinetics and disease severity (Results: Line 285-292). We found two sublineages of DENV3-IIIA co-circulating in all regions of the country (Results: Line 299-300). Separately, the dramatic shift from lineage Ic to Id observed for DENV4, is described in detail in our manuscript (Results: Line 310-314). Based on the available DENV4 sequences DENV4-Id has replaced DENV4-Ic in South and Central India. The emergence of lineage-Id also coincided with the 2017 outbreak in South India and has replaced DENV4-Ic lineage.

In North India, we observed a series of outbreaks (2006, 2010, 2013) with a different serotype dominating during each outbreak suggesting a pattern of serotype replacement (Results: Line 222-227). However, in South India, we continue to observe all the serotypes over the last two decades.

A critical observation from the dynamics of dengue in India points to an interplay between the population immunity and virus convergence to 'related' E gene variants. We observe several signatures of these in our manuscript including showing how DENV4-Id lineage has gained mutations pushing it closer to DENV1/3 (Results Line: 345-354). If indeed antigenic forces are driving such dynamics, we expect to see stronger indicators of coupling between the dengue serotypes as seropositivity in India increases in coming years.

Reviewer #3: In this paper, the authors explore the genetic diversity of dengue viruses in India. They compare the sequences of the circulating viruses with that of vaccines, demonstrating that they are quite different in antigenically relevant sites. India probably suffers from the greatest burden of dengue infection worldwide, but there have been few studies that have characterised the genetic diversity in the country. This study is therefore welcome. I have a few comments.

We appreciate the reviewer's comments and thank them for highlighting the importance of our work. We have provided point-by-point responses to the queries raised below.

Part III – Minor Issues: Editorial and Data Presentation Modifications

Please use this section for editorial suggestions as well as relatively minor modifications of existing data that would enhance clarity.

Reviewer #1: The latter section of the introduction appears to give a summary of all the results. I would suggest removing this section from here and putting in the discussion. Some of the comments below are on this section and these will hold when the section is moved to discussion.

We thank the reviewer for making this suggestion. We have shifted this part of the introduction to the discussion section now as suggested by the reviewer. We believe this rearrangement makes this part more clear and provides the context for the discussion that follows.

Line 84-85: What do you mean by serotype replacement?

Serotype replacement is commonly identified as an event when a dominant dengue serotype is replaced during a subsequent outbreak by another serotype. Commonly, this is mainly attributed to the differential protection provided by a primary dengue infection against different serotypes. In the case of homotypic secondary infection (same serotype as the primary infection) protection lasts for a substantially longer period (order of a decade), while in the case of heterotypic infection (a different serotype than the primary infection) protection wanes within 2-4 years. This reduced immunity against a distinct second serotype may allow the serotype to manifest an epidemic outbreak (with a gap of 2-4 years). Such serotype replacement events of course depend on the prevalence of serotypes, population immunity in the region and antigenic proximity to the prior infecting serotype.

We have elaborated this point in the manuscript as below.

Introduction: Original statement (Line 81-82)

Cyclic dengue outbreaks in the endemic regions occur every 2-4 years, often associated with serotype/genotype replacement [18–21].

Introduction: Revised statement (Line 81-86)

Cyclic dengue outbreaks in the endemic regions occur every 2-4 years, often associated with serotype/genotype replacement, where serotype/genotype dominance changes during the subsequent outbreak [18–21]. This has been attributed to a combination of long-term protection from homotypic dengue infection albeit short-term protection (up to 3 years) from the heterotypic secondary infection [22–24]. This allows the heterotypic serotype to manifest an outbreak depending on the prevalence of serotypes and population immunity in the region [18].

Could you be more specific about how ADE plays a role in increasing dengue infections (eg increasing from what, and is it infections or cases?)

During the secondary heterotypic infection, the role of antibody dependent enhancement (ADE) in enhancing the disease severity by increasing the viremia is well reported *in-vivo* (Goncalvez

et al., 2007; Halstead, 1979) and *in-vitro* (Lin et al., 2002; Quinn et al., 2013; Yamanaka et al., 2021). Higher viremia is also linked with higher transmission to mosquitoes (Duong et al., 2015; Nguyet et al., 2013) thus increasing the number of dengue cases. Hence, we argue that prevalence of dengue virus strains that display ADE properties can lead to increased transmission. We have elaborated and explained this idea in the revised version of the manuscript.

Introduction: Original statement (Line 84-86)

During serotype replacement, ADE can also play a role in increasing dengue infections, depending on the level of cross-reactive immunity in the population, thereby making the cyclic pattern of outbreaks more prominent.

Introduction: Revised statement (Line 86-89)

During serotype replacement, ADE can also play a role in increasing dengue cases. Increased dengue viremia levels observed during ADE [25–27] can contribute to increased transmission of the virus [28,29]. Therefore, ADE related effects can further make the cyclic pattern of outbreaks more prominent.

Line 114: I assume other sequences have been brought in for this part of the analysis. Please give a bit more detail on where this result is from.

Yes, all the publicly available whole genome dengue sequences from the ViPR database were used for this analysis (Dataset B). We have moved this part to the discussion which helps since the results and methods now discuss this first providing the context and details.

Introduction: Original statement (Line 114)

The spatio-temporal analysis shows broad geographical restriction of the Indian genotypes to Asia.

Discussion: Revised statement (Line 501-502)

Phylogenetics analysis using the global dengue virus sequences also highlights the geographical restriction of dengue viral diversity (Fig 2, S7 Fig).

Line 116-118: What is the interpretation of the result about Hamming distances? Is this linked to the comment about cross reactive immunity? It currently isn’t clear- suggest rephrasing.

We measured the divergence in the dengue viruses over the years and between the serotypes using the hamming distances. We found a good correlation between the Hamming distances and antigenic titer data available from other studies (This is now included in a new figure; S9 Fig). Thus, we employ the Hamming distance fluctuations as a proxy of antigenic distances.

Usage of Hamming distance and the interpretation with the cross-reactive immunity is now discussed in detail in the manuscript (Results: Line 362-366). We have also shifted this sentence from the introduction to the discussion section to further reduce the confusion.

Line 120: What is the "this" that has lead to the highly divergent DENV4 and what is the evidence that "this" has caused it? Why just for DENV 4?

In South India, DENV1/2/3 were the dominating serotypes until 2015 while DENV4 prevalence became significant thereafter. We show that the DENV4 sequences are becoming more similar to DENV1 and DENV3 E gene sequences (Fig 3). We postulate that this DENV4 drift towards DENV1/3 is driven by underlying population immunity (seroprevalence of ~75%) (Murhekar et al., 2019). Pre-exisiting immunity to DENV1/2/3 prevents corresponding outbreaks but can promote increased transmission of DENV4 variants that can utilize cross-reactive antibodies from prior infections through ADE. This can be one of the driving forces for the evolution of DENV4 into a new lineage that mimics DENV1/3.

We have shifted this part to the discussion section where it will be easier to follow our argument after several lines of evidence are presented in the results section regarding this point.

Introduction: Original statement (Line 120-121)

This has led to the emergence of a highly divergent DENV4 lineage in India, with evidence of strong immune selection pressure on the E gene.

Discussion: Revised statement (Line 487-494)

In South India, DENV1/2/3 were dominating serotypes till 2015, and DENV4 emerged as one of the dominant serotypes in South India thereafter (Fig 1B) [33,65]. Most of the divergence in the Indian DENV4-Id lineage is largely restricted to immunodominant E and NS2A proteins, again pointing to immune evasion driving its emergence. Consistent with our observation of interserotype correlations, we observed this lineage is moving towards DENV1 and DENV3 serotypes. Prior seropositivity due to exposure to DENV1/2/3 prevents new outbreaks by these serotypes but enhanced similarity of DENV4-Id to these serotypes might contribute to ADE mediated increased transmission during the secondary infection with DENV4 in the Indian population.

Figure 1: Are there any differences between the two directions of the y axis 0-100? Suggest updating legend to explain or editing figure so that is is more easily interpretable.

There is no difference between the two directions of the y-axis. We agree that the original figure had a confusing y axis. Therefore, we have modified Figure 1 in the revised manuscript

(reproduced below). We hope now it is easy to interpret.

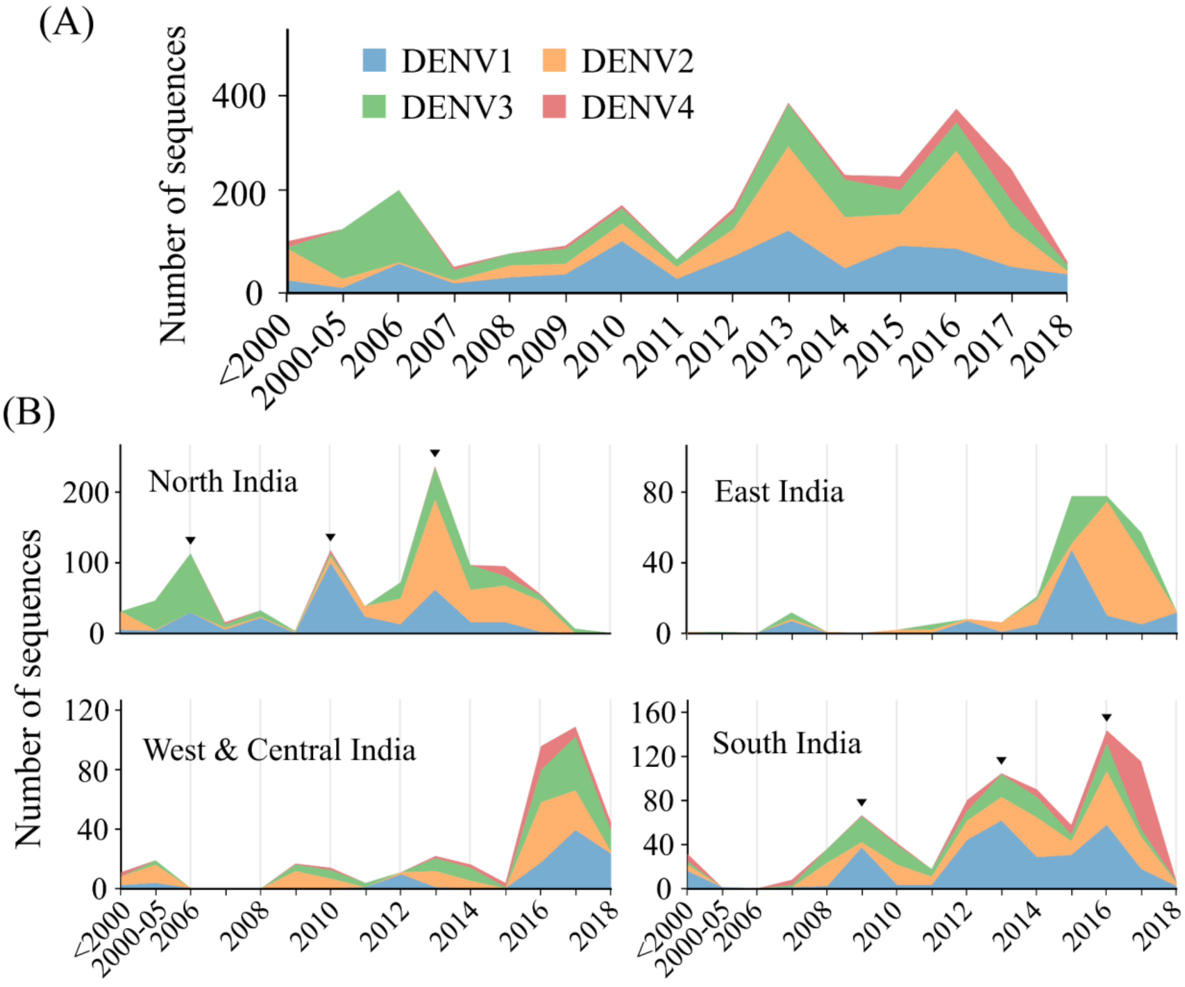

Fig 1. Year-wise dengue sequences reported from India.
(A) Dengue serotype sequences available from India till 2018. (B) Distribution of dengue serotypes in different regions of India. DENV1: blue, DENV2: orange, DENV3: green, DENV4: red. Arrows indicate the peaks in the number of sequences.

Subset C: It isn't clear from the description if this dataset is sequences from India, or all sequences of those found in India (but including elsewhere). Suggest rephrasing.

To investigate how the genotypes from India are involved in import and exports from other countries, we included the global sequences which belong to the Indian genotypes for our spatio-temporal analysis. We have explained this in the revised manuscript.

We have also renamed Subset C to Dataset B.1 and Dataset D to Dataset C to indicate this. List of the accession numbers along with the location and date of collection is provided in the Supporting Table 1.

Methods: Original statement (Line 136-138)

Subset C: For analysis of spatio-temporal dynamics, protein-coding sequences specific to the Indian genotypes were selected from dataset B. We included all unique protein-coding sequences from India and randomly sampled global sequences.

Methods: Revised statement (Line 129-132)

Dataset B.1: For analysis of spatio-temporal dynamics, protein-coding sequences specific to the Indian genotypes were selected from dataset B. We included all unique protein-coding sequences from India and augmented the set with 362 randomly sampled global sequences belonging to the Indian genotypes (S1 Table).

Why was 2019 removed for COVID?

We assigned peaks in the number of cases based on the number of cases in the previous year. (more than 50% increase from the previous year's number of cases). Since the 2020 data was incomplete due to COVID-19-related disruptions, we avoided using the number of cases from 2019 to check if there was any peak during 2020. We have added this point in the revised version.

Methods: Original statement (Line 145-146)

The cases from 2019-2022 were excluded while considering the spikes due to the effect of COVID-19-related disruptions [36].

Methods: Revised statement (Line 139-142)

Peaks in the number of cases and deaths were assigned for a particular year if the number of cases/deaths in that year increased more than 50% from the previous year. Due to the COVID-19-related disruptions from 2020 onwards, the cases from 2019-2022 were excluded for determining the peaks [41].

Line 236-237 and Line 242- 243: Appears to be a repetition of the DENV4 emergence in South Indian result.

The last statement is summarizing the general trends we see in the dengue serotype dynamics in India. We have clarified this in the revision.

Results: Original statement (Line 240-242)

Overall, in India, DENV1 and DENV3 were the dominating serotypes till 2012. DENV2 has become the dominant serotype in most regions in India since then, and DENV4 is establishing itself in South India.

Results: Revised statement (Line 238-240)

In summary, DENV1 and DENV3 were the dominating serotypes in India till 2012. DENV2 has become the dominant serotype in most regions in India since then, and DENV4 is establishing itself in South India.

Line 253 -255: Spatially within India or globally

Although we found one genotype under each serotype in India (except for DENV1), we found multiple lineages/ sublineages under each genotype propagating in the country. Many lineages are co-circulating in India and sometimes in the same region. The rest of the paragraph in the manuscript proceeds to discuss this diversity in dengue genotypes in the revised version with more details. Hopefully, this removes the ambiguity indicated by the reviewer.

Results: Original statement (Line 253-254)

Interestingly, we find distinct temporally and spatially co-circulating lineages within the genotypes (Fig 2B-D), yet it remains unclear what factors contribute to their co-prevalence.

Results: Revised statement (Line 251-252)

Interestingly, we find distinct temporally and spatially co-circulating lineages within the genotypes in India (Fig 2B-E), yet it remains unclear what factors contribute to their co-prevalence.

For the global comparisons it would be helpful to have some idea of the numbers of sequence available for different countries. It is currently hard to interpret whether these genotypes are known to not circulate in some countries or if there just isn't much data.

This data was based on all the whole genome dengue sequences (n = 4238) available worldwide. Fig 2A shows the geographical clustering of genotypes at the continental or sub-continental level. It is not surprising that some countries are under-represented due to poor sequencing efforts in their respective regions. Therefore, we restrict ourselves to discussing these trends at the continental level. In spite of this, the geographic localization of dengue genotypes at the continental level is quite evident as shown in Fig 2A.

For the spatio-temporal spread analysis, we have used Dataset B.1, where sequences belonging to Indian genotypes were randomly sampled from across the world (160 Indian sequences combined with 362 sequences from other countries). The list of the accession numbers, location and date of collection of the sequences included in the analysis is provided in the revised S1 Table.

Methods: Revised statement (Line 129-134)

Dataset B.1: For analysis of spatio-temporal dynamics, protein-coding sequences specific to the Indian genotypes were selected from dataset B. We included all unique protein-coding sequences from India and augmented the set with 362 randomly sampled global sequences belonging to the Indian genotypes (S1 Table). This included a total of 522 sequences from DENV1 genotype I (n = 142), DENV1 genotype III (n = 93), DENV2 genotype Cosmopolitan (n = 144), DENV3 genotype III (n = 96), and DENV4 genotype I (n = 47).

Revised S1 Table (Line 935-937)

S1 Table. Accession Numbers for all datasets

A list of accession numbers for Dataset B, B.1 and C is given. Dataset B.1 also includes the place and year of collection of the corresponding dengue sequencing sample.

It would be helpful to state what is the evidence for the temporality inferred from the trees?

Temporality inferred from the tree is based on the year when the samples were collected. We performed Bayesian analysis using BEAST v1.8.3 to estimate the import-export period and the year of common ancestors. This is described in the methods section (Line 156-159)

Line 418-419: Please cite a reference for these regions with variations as having a positive antigenic impact or epitopic regions.

Thanks for pointing out the missing reference. We have now cited the reference in the revised version. We selected the experimentally validated epitopic regions available from the IEDB database as described in the methods section (Line ~193-200). Sites with positive antigenic effects were included from a recently published study (Huang et al., 2022).

Results: Original statement (Line 418-419)

Further, almost half (34.6-66.7%) of all the E protein variations lie either in known epitopic regions or have a positive antigenic effect.

Results: Revised statement (Line 420-421)

Further, almost half (34.6-66.7%) of all the E protein variations lie either in known epitopic regions selected from the IEDB database or have a positive antigenic effect [52] (S7 Table).

I find the conclusions about the impact of immune selection on the viruses interesting, but I'm not completely sure how it is supported by the data- be interested to have more discussion of this.

Indeed, the interaction between population immunity and viral dynamics has been less studied, and signatures of dengue virus evolution under immune selection pressure has not been reported from India. It has been speculated that high seroprevalence in a population can drive virus evolution which is especially evident in the immunogenic viral proteins (Pybus & Rambaut, 2009). Host immunity is also considered as a major selection pressure driving the evolution of the SARS-CoV-2 virus in the last two years (Cai et al., 2021; Rubio-Casillas et al., 2022).

Our study presents several lines of evidence for dengue evolution in India being strongly influenced by population immunity. First, we found multiple lineages/sub-lineages of the DENV serotypes in India. Among these lineages, several of the E gene differences were in the known epitopic regions, suggesting the role of immune selection pressure leading to the emergence of potential 'immune escape' variants. Although we do not provide measurements of antibody

neutralization titers in our manuscript, we have discussed prior studies (Results section: Line 432-443) where some of these residues have been linked to the reduction in antibody neutralization titers.

Second, we found strong evidence of such immune selection pressure on DENV4 in India, consistent with significantly higher dN/dS values (Fig 3D) for the immunogenic envelope gene. The Indian lineage DENV4-Id has evolved from DENV4-Ic in a short time frame with about 30% mutations lying in the E gene (S5D Fig). Moreover, half of these E gene mutations were identified in antigenic regions. More interestingly, DENV4-Id has acquired residues that make it similar to DENV1 and DENV3. With about ~75% seropositivity in South India, most of the population has already developed antibodies against DENV1/2/3 that dominated infections in India between 1980-2012. Thus, we speculate that DENV4 has acquired immune epitope signatures comparable to DENV1/2/3 serotypes to gain advantage via ADE as discussed in the manuscript (Discussion section: Line 485-492).

Third, while all major dengue vaccines are derived from strains outside India, comparison with E gene structure shows that majority of the differences in Indian lineages lie on the antibody accessible outer surface of the virus particle. In fact, 3-13% of all the known E protein epitopic sites are mutated in Indian dengue strains. Further, about half of differences between the vaccines and the Indian dengue variants in the E protein are localized to immunogenic residues (S7 Table). These points are discussed in the Results section: Line 416-424.

We have strengthened our discussion of these points in the revised manuscript by including the following in the Discussions.

Discussion: Revised statement (Line 487-494)

In South India, DENV1/2/3 were dominating serotypes till 2015, and DENV4 emerged as one of the dominant serotypes in South India thereafter (Fig 1B) [33,65]. Most of the divergence in the Indian DENV4-Id lineage is largely restricted to immunodominant E and NS2A proteins, again pointing to immune evasion driving its emergence. Consistent with our observation of interserotype correlations, we observed this lineage is moving towards DENV1 and DENV3 serotypes. Prior seropositivity due to exposure to DENV1/2/3 prevents new outbreaks by these serotypes but enhanced similarity of DENV4-Id to these serotypes might contribute to ADE mediated increased transmission during the secondary infection with DENV4 in the Indian population.

Discussion: Revised statement (Line 506-513)

Moreover, most of the variations from vaccine strains are concentrated on the exposed regions of the E protein, including the EDIII region, which plays a significant role in defining the antigenicity [84] and is the primary target of the neutralizing antibodies [85,86]. Therefore, an in-depth characterization of the effect of the reported differences on the antibody titers is required to assess the efficacy of prospective dengue vaccines for India. One can argue that regionally tailored vaccines would be more efficacious due to the geographical restriction of dengue [83].

However, the impact of immunity developed by vaccination on the future course of dengue virus evolution needs to be considered carefully.

Reviewer #2: Inconsistency between the legend (line 519) and Figure 2 B; DENV-1 III or I?

Thanks for pointing it out. It should be DENV1-III. We have corrected this in the revision.

Original statement (Line 519-520)

(B-E) Time-dated phylogenetic trees for all serotypes with the circulating genotypes in India (DENV1-I, DENV2-cosmopolitan, DENV3-III, DENV4-I).

Revised statement (Line 530-531)

(B-E) Time-dated phylogenetic trees for all serotypes with the circulating genotypes in India (DENV1-III, DENV2-cosmopolitan, DENV3-III, DENV4-I).

Reviewer #3: Page 6 – I believe it should say 'Dataset C' and not 'Subset C'

Set C does not contain any new sequences; it is actually a subset of Dataset B. Therefore, we have renamed Subset C to Dataset B.1 to avoid further confusion. We have made the required changes in all the places where these datasets are mentioned.

Methods: Revised statement (Line 129-132)

Dataset B.1: For analysis of spatio-temporal dynamics, protein-coding sequences specific to the Indian genotypes were selected from dataset B. We included all unique protein-coding sequences from India and augmented the set with 362 randomly sampled global sequences belonging to the Indian genotypes (S1 Table).

Part II – Major Issues: Key Experiments Required for Acceptance

Please use this section to detail the key new experiments or modifications of existing experiments that should be absolutely required to validate study conclusions.

Generally, there should be no more than 3 such required experiments or major modifications for a "Major Revision" recommendation. If more than 3 experiments are necessary to validate the study conclusions, then you are encouraged to recommend "Reject".

Reviewer #2: - Authors should clearly describe the sample size's representative-ness.

We understand the reviewer's concern regarding the representativeness of our sample size. For example, between 2017-19, roughly 15000 dengue cases were reported yearly from India, while only about 300 dengue genomic sequences are reported annually. It is clearly a small representation of the total incidences (2%). Further, in absence of epidemiological metadata associated with the submitted virus sequences, it is also difficult to ascertain the representativeness in terms of clinical spectrum or demographics. We have elaborated this issue below.

Data representative-ness for prevalence and evolution:

1. Bulk of the dengue cases are reported from urban centers in India. Similarly, the genomic sequences are also mostly limited to urban areas which are accessible to researchers. This skews the geographical representation of dengue virus genomes but is otherwise representative of corresponding caseloads.
2. Based on the global sequences, we see that the dengue spread is restricted to continental or sub-continental boundaries. Moreover, only one genotype under each serotype dominates in India. Minor variants of the virus might be present in India and missed in our analysis but this does not alter our findings significantly.
3. Due to changing serotype distribution every year, it is hard to get equal representation of all the serotypes uniformly across the years. Nevertheless, serotype distribution from a recent nation-wide study from India ((Alagarasu et al., 2021), DENV1 32%, DENV2 33%, DENV3 28% and DENV4 7%) matches serotype distribution in our dataset A (DENV1 32%, DENV2 37%, DENV3 26% and DENV4 4%). Also, the data is not biased towards the year of collection, since we see a good correlation between annual number of cases and number of sequences reported yearly (Fig S1E; Pearson's correlation coefficient = 0.67). (Results section: Line 217-220)
4. To get the substitution rates, we have randomly selected a subset of sequences and estimated the rates independently for 5 runs effectively randomizing our dataset. Our substitution rates also match previously reported numbers from other studies for all the serotypes (Results section: Line 156-159).

Data representative-ness of dynamics:

1. To ensure that the correlations observed among dengue yearly variants are not arising due to the ‘noise’ in small sample sizes, we employ multiple bootstrapping based strategies as described in the methods section (Line 186-192).
    a. Bootstrapping with deleting up to 3 time points does not remove the correlations
    b. Bootstrapping by selecting individual traces randomly still displays similar correlations
    c. Randomly shuffling the time series to ensure that the correlations do not arise merely from the yearly fluctuations

    With all these ‘controls’, we argue against these correlated fluctuations are merely arising from noise in ‘randomized’ sets. This suggests that dengue evolution in India is indeed ‘correlated’.

2. To check if the fluctuations are specific to the immunological E gene, we also performed the same analysis for the CprM gene and did not find any evidence of fluctuations at both inter- and intra-serotype dynamics. This suggests that immunological pressure is probably the underlying driving factor giving rise to such correlated fluctuations in acquired mutations.

Therefore, despite some of gaps discussed above, our findings are representative of dengue evolution in India as well as population immunity being a major factor in the dynamics observed. Further, our analysis focuses on all currently available dengue virus sequences. In the absence of larger datasets, we have relied only on direct comparisons among the available genome sequences from India. We estimate the detection of dengue variants at ~ 5% prevalence with a 95% confidence interval (with finite population size correction) for our data analysis. Here, the evolutionary dynamics trends reported can be attributed above this detection threshold based on the comparison of related genotypes in our study.

We have discussed the limitations of data-representativeness and other limitations explicitly in the corresponding results sections of the revised manuscript. Those are reproduced for the reviewer’s perusal below.

Regarding the correlation between the cases and number of sequences (Results section: Line 217-220)

Although dengue sequence reporting from various parts of the country is sporadic and focused on the urban areas, the number of annual cases and deaths in the past two decades correlated well with the number of available sequences each year (S1E Fig, Pearson’s correlation coefficient = 0.67).

Regarding the substitution rates (Methods section: Line 156-159)

Bayesian analysis was performed with the E gene, NS5 gene and whole-genome sequences from dataset B.1 using the BEAST v1.8.3 [42] to get the substitution rates for each gene. Five sets were generated for each serotype by selecting 80% sequences randomly, the substitution rate was calculated for each run, and the average substitution rate was reported.

Regarding the bootstrapping in the evolutionary dynamics (Methods Line 186-192)

Pearson's correlation coefficient was obtained between the serotype and inter-serotype distance dynamics. The robustness of the correlations was checked by three methods. First, by randomly deleting up to 3 data points from each dynamics (1000 bootstraps). Second, by estimating the cross-correlation between individual traces from two comparing groups randomly with 1000 bootstraps. As a control, we also checked correlations between the groups by randomly shuffling the time series of normalized hamming distances (before correlating) to ensure that the correlations do not arise merely from the yearly fluctuations (1000 bootstraps).

Regarding CprM gene

Methods: Revised statement (Line 183-185): We performed the same analysis with the CprM gene sequences (position 55 to 155 of the coding sequence) from South Indian sequences (n=411).

Results: Revised statement (Line 390-393): These signatures are specific to the immunologically dominant E gene. For example, when we compare the CprM gene sequences from South India, the fluctuations were not significant. When CprM Hamming distance fluctuations are observed as in the case of D1 dynamics, it is linked to the transient introduction of genotype I in 2012 (S11A Fig).

- Manuscript should include a more detail epidemiological data and explain whether there is direct correlation of specific genotype/lineages with the outbreaks.

We have discussed this aspect in the part I of the review, we are reproducing the response here for the convenience of the reviewer.

We agree with the reviewer that the clinical and epidemiological data would strengthen our observations and provide new insights that are even beyond the current manuscript. In the current study, we have tried to comprehensively cover all available genomic data from the Genbank and/or ViPR database. Unfortunately, clinical metadata is not available corresponding to many of the Indian virus sequences. This limits our ability to draw causal inferences only regarding genetic evolution of the virus in India with broad generalizations.

Mitigation of future epidemics is a key motivation of our work. However, prediction of future epidemics requires coordinated efforts to incorporate studies like ours in better multi-parametric models. Such models must incorporate and analyze data on factors that influence dengue transmission, population immunity, virus evolution, climate, population density, and urbanization among others. This is unfortunately beyond the scope of our current manuscript but will be part of future work.

Inspite of these limitations, our manuscript discusses several epidemiological insights including lineage/sublineage changes, co-incidence of outbreaks with serotype replacement events, emergence of new strains, co-circulation of closely related genotypes and the dynamics of genetic changes in the Indian dengue strains in the results section.

DENV1 genotype III, the dominant genotype in India has two lineages (lineage-I and II) co-circulating in the country (Results: Line 253-258). While DENV1-III lineage-I is present across the country, lineage-II is circulating only in South India (Results: Line 266-269). A distinct DENV1-I genotype was introduced in South India briefly between 2012-15 but not reported since. For DENV2, the cosmopolitan lineage I and III are reported extensively from across the country. Our group has previously shown that the replacement of lineage-I by lineage III in New Delhi even though they don't vary in terms of growth kinetics and disease severity (Results: Line 285-292). We found two sublineages of DENV3-IIIA co-circulating in all regions of the country (Results: Line 299-300). Separately, the dramatic shift from lineage Ic to Id observed for DENV4, is described in detail in our manuscript (Results: Line 310-314). Based on the available DENV4 sequences DENV4-Id has replaced DENV4-Ic in South and Central India. The emergence of lineage-Id also coincided with the 2017 outbreak in South India and has replaced DENV4-Ic lineage.

In North India, we observed a series of outbreaks (2006, 2010, 2013) with a different serotype dominating during each outbreak suggesting a pattern of serotype replacement (Results: Line 222-227). However, in South India, we continue to observe all the serotypes over the last two decades.

A critical observation from the dynamics of dengue in India points to an interplay between the population immunity and virus convergence to 'related' E gene variants. We observe several signatures of these in our manuscript including showing how DENV4-Id lineage has gained mutations pushing it closer to DENV1/3 (Results Line: 345-354). If indeed antigenic forces are driving such dynamics, we expect to see stronger indicators of coupling between the dengue serotypes as seropositivity in India increases in coming years.

- Whether the data presented can be used to predict future outbreak should be discussed.

Currently, there are no robust models that predict future outbreaks of dengue or other flaviviruses. Existing attempts at such models have several limitations especially in predicting the future outbreaks. We believe incorporating the interactions between the serotypes as identified in our study into the models can help in this regard. We are currently working on developing a model considering these interserotypic 'interactions' within the context of an underlying population seropositivity. However, such work requires extensive datasets of prevalent strains, region-wise seropositivity, prior infecting serotypes and other anthropogenic characteristics. We are currently working on such models but it is too premature to make claims about their ability to predict future outbreaks here.

Discussion: Revised statement (Line 516-517)

Incorporating such cross-serotype virus-host immunological interactions can improve dengue epidemiological modelling in the future.

- The differences of the vaccine strains with India's strains should be compared with other countries in terms of possible low efficacy caused by genotype differences.

We show that out of 12 strains from 3 commercial vaccines that we considered, only 1 strain represents the genotype circulating in India over the last 20 years. Such antibody neutralization and vaccine efficacy studies for Indian viruses have not been conducted till date. Therefore, we have relied on comparison of vaccine efficacy from other countries against different strains to draw our conclusions.

The genotype differences can cause significant drop in antibody titers and vaccine efficacies (Bell et al., 2019; Juraska et al., 2018; Martinez et al., 2020). We have discussed this aspect in our manuscript especially in the context of the mutations observed in Indian dengue variants (Line 432-443). Briefly, previous studies have shown up to two-log reduction in neutralization antibody titers with different genotypes (D. et al., 2010; Martinez et al., 2020). Similarly, human and macaque challenge studies (Kirkpatrick et al., 2016; Osorio et al., 2011) showed complete protection against the infection against the vaccine genotype while only partial protection against infection with a different genotype. We have also discussed a mutational sieve analysis for DENV4 vaccine (Juraska et al., 2018) which shows reduced vaccine efficacy caused due to differences in genotypes.

Results: Revised statement (Line 431-434)

Therefore, antigenic differences of Indian dengue viruses with respect to the vaccines define the majority of E protein differences. This can have important implications for vaccine efficacy, where vaccines will likely evoke poor neutralization against dengue virus in India.

- Limitations should be clearly described.

The points and limitations discussed above have been now included in the results and discussion section. Please refer to the highlighted sections in the results and discussion section of the revised manuscript. We have reproduced it here for the convenience of the reviewer.

Revised statements regarding data representative-ness

Regarding the correlation between the cases and number of sequences (Results section: Line 217-220)

Although dengue sequence reporting from various parts of the country is sporadic and focused on the urban areas, the number of annual cases and deaths in the past two decades correlated well with the number of available sequences each year (S1E Fig, Pearson’s correlation coefficient = 0.67).

Regarding the substitution rates (Methods section: Line 156-159)

Bayesian analysis was performed with the E gene, NS5 gene and whole-genome sequences from dataset B.1 using the BEAST v1.8.3 [42] to get the substitution rates for each gene. Five sets were generated for each serotype by selecting 80% sequences randomly, the substitution rate was calculated for each run, and the average substitution rate was reported.

Regarding the bootstrapping in the evolutionary dynamics (Methods Line 186-192)

Pearson’s correlation coefficient was obtained between the serotype and inter-serotype distance dynamics. The robustness of the correlations was checked by three methods. First, by randomly deleting up to 3 data points from each dynamics (1000 bootstraps). Second, by estimating the cross-correlation between individual traces from two comparing groups randomly with 1000 bootstraps. As a control, we also checked correlations between the groups by randomly shuffling the time series of normalized hamming distances (before correlating) to ensure that the correlations do not arise merely from the yearly fluctuations (1000 bootstraps).

Regarding CprM gene

Methods (Line 183-185): We performed the same analysis with the CprM gene sequences (position 55 to 155 of the coding sequence) from South Indian sequences (n=411).

Results (Line 390-393): These signatures are specific to the immunologically dominant E gene. For example, when we compare the CprM gene sequences from South India, the fluctuations were not significant in any case apart from D1 dynamics but that is linked to the introduction of genotype I in 2012 (S11A Fig).

Reviewer #3: The largest limitation in the paper is that the authors make strong conclusions about the antigenic evolution of the serotypes, however, there is no direct measurement of antigenicity (such as neutralisation). Instead, they rely on the presence of nucleotide changes in previously identified epitopes, without being able to characterise what those changes actually do to neutralisation (if anything). I would suggest the authors discuss this limitation, and tone down the language when making conclusions about antigenic evolution.

We agree with the reviewer that we do not have antigenicity data for the Indian dengue variants and we hope to rectify this in future. For our current analysis, we rely on correlative evidence that suggests that differences in E protein residues can be representative of dengue antigenic differences. Bell *et al.* have shown that the dengue antigenic evolution corresponds to the E gene divergence (Bell et al., 2019).

Further to test correlation between E gene mutations and antigenic distances, we compared the antigenic titers between different viruses to the similarity between the E gene amino acid sequences (Pearson’s correlation coefficient = 0.53) (now included as a new supplementary figure in revised manuscript, S9 Fig). Antigenic titers are based on the neutralization antibody titers collected from humans (post monovalent vaccination) and African green monkeys (post-infection with 16 viruses) against a panel of 36/37 dengue representative viruses (Katzelnick et al., 2015). This suggests that dengue E gene variations are generally correlated with virus antigenicity. Therefore, in absence of antigenicity data, we have employed hamming distance variations in E gene to argue for generalized trends in antigenic evolution. In the future, we hope our datasets are refined further with dengue antigencity data and accurate models that predict virus antigenicity from amino acid changes.

We have added this argument to the results and discussion sections in the revision and modified the text to tone down our inferences.

Revised statement in Results (Line 360-366)

In the absence of antigenicity data, we evaluated the variation in amino acid sequences of the E gene longitudinally. To validate this approach, we rely on neutralization antibody titers of sera collected from humans (post-monovalent vaccination) and African green monkeys (post-infection) tested against a panel of viruses [72]. We found general correlation between the antibody titers and the similarity between the E protein sequences with the two datasets of antibody titer data (Pearson's correlation coefficient: 0.530, S9 Fig).

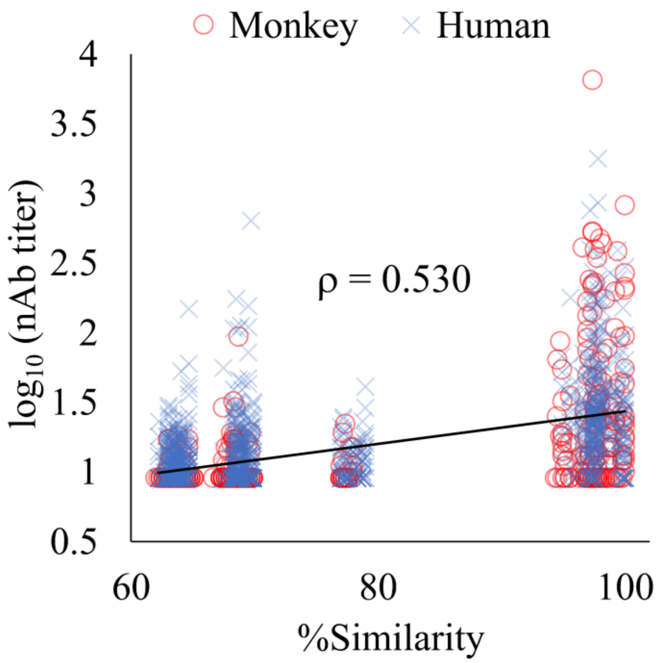

New figure legend (Line 898-902)

S9 Fig. Sequence similarity and neutralization antibody titer.

Correlation between neutralization antibody titer collected from humans (x) collected post-vaccination and African green monkeys (o) collected post-infection and tested against a panel of viruses. The X-axis denotes the percentage similarity between the amino acid sequences of the infecting and test viruses.

The last sentence in the abstract concludes that the high incidence and pre-existing immunity are shaping dengue evolution. However, there was no attempt to reconstruct population immune profiles in the paper, so I'm not sure the authors can really conclude this.

We agree with the reviewer that we do not have direct evidence of pre-existing immunity driving the viral evolution. In-vitro evolution studies coupled with detailed population immune profiles of dengue antigenicity can help understanding certain aspects of this. We have revised the abstract to reflect this.

We would also like to point out that many threads of evidence suggest that population immunity might be at play.

1. High seropositivity (~75%) reported in south India in a recent survey (Murhekar et al., 2019) suggesting pre-existing dengue immunity. Recent emergence of DENV4 (2015) in regions previously endemic to DENV1/3 and acquisition of DENV1/3 E gene mutations by the emergent DENV4-Id variant
2. Correlated fluctuations among the serotypes with a time period of ~ 3 years that also is comparable to decay in cross-reactivity antibodies.
3. Co-incidence of serotype replacement and regional outbreaks.

We hope future studies will investigate this interdependence in detail.

Revised statement (Line 42-43)

Our study shows how the dengue virus evolution in India is being shaped in complex ways.

A major conclusion from the paper is that the genetic distance (as measured by Hammings distance) between the E-gene of strains has fluctuated with 3-5 year periodicities. The authors conclude this is linked to population immunity changes. I think this is an interesting observation but I'm not sure the authors can conclude that these fluctuations are due to immunity. To better assess a potential role of immunity, the authors could repeat the analysis with the rest of the genome. If the changes in the E-gene were really driven by immunity, these fluctuations would be absent in parts of the genome that are not antigenically relevant.

We agree. Indeed, we argued this point with the same reasoning and associated analysis. In Figure S10A of the original manuscript, we show this analysis based on the CprM gene. As expected for non-immunogenic genes, we did not observe strong fluctuations in this part of the genome unlike the E gene, except for the DENV1, but that could be due to the presence of genotype I in 2012 (S10 Fig). Non-structural genes would have been an ideal choice for this analysis but currently we do not have sufficient number of whole genome sequences from India to conduct this analysis.

Methods: Revised statement (Line 183-185)

We performed the same analysis with the CprM gene sequences (position 55 to 155 of the coding sequence) from South Indian sequences (n=411).

Original statement is reproduced below (Line 390-393)

For example, when we compare the CprM gene sequences from South India, the fluctuations were not significant in any case apart from DENV1, but that is linked to the introduction of genotype I in 2012 (S11A Fig).

On page 13 the authors say that DENV4-1 originated from the Philippines. I would argue this is very difficult to conclude as there are very few DENV4 sequences available from the region and it is very possible that the origin is in an unsampled location.

Based on the currently available sequences, DENV4-1 was originally identified in Philippines. A similar analysis using the E gene sequences also reports the same origin (Sang et al., 2019). It is still possible that the origin could have been in a place that is difficult to ascertain. We have rephrased the statement accordingly.

Original statement (Line 306-307)

The spatio-temporal reconstruction identifies the origin of DENV4-I in the Philippines (S7C Fig), consistent with Sang et al. [59].

Revised statement (Line 304-305)

The spatio-temporal reconstruction identifies the earliest sequences of DENV4-I of Philippines origin (S7C Fig) consistent with the E gene analysis reported earlier [64].

I found Figure 4C very difficult to read/interpret. I'm afraid I don't have a better suggestion but anything that helps guide the reader interpret the plot would be useful.

We have modified the figure based on the reviewer's comment. In the revised Fig 4C, we now report the median of Pearson's correlation coefficient (PCC) obtained from the histogram obtained using the bootstrapping methods. Here, PCC is a measure of the correlated movement of the dengue E gene in the sequence space. The complete histogram of correlation coefficients (previous version of Fig 4C) is reproduced in S10 Fig in the revised manuscript for the benefit of the reader. Further, to clarify the essence of the positive (or negative) correlations between D_{ij} and D_i or D_j, a schematic with the directional vectors depicting the within and between serotypic evolution is shown in Fig 4D. The new figure and legends are reproduced below.

Revised Figure 4

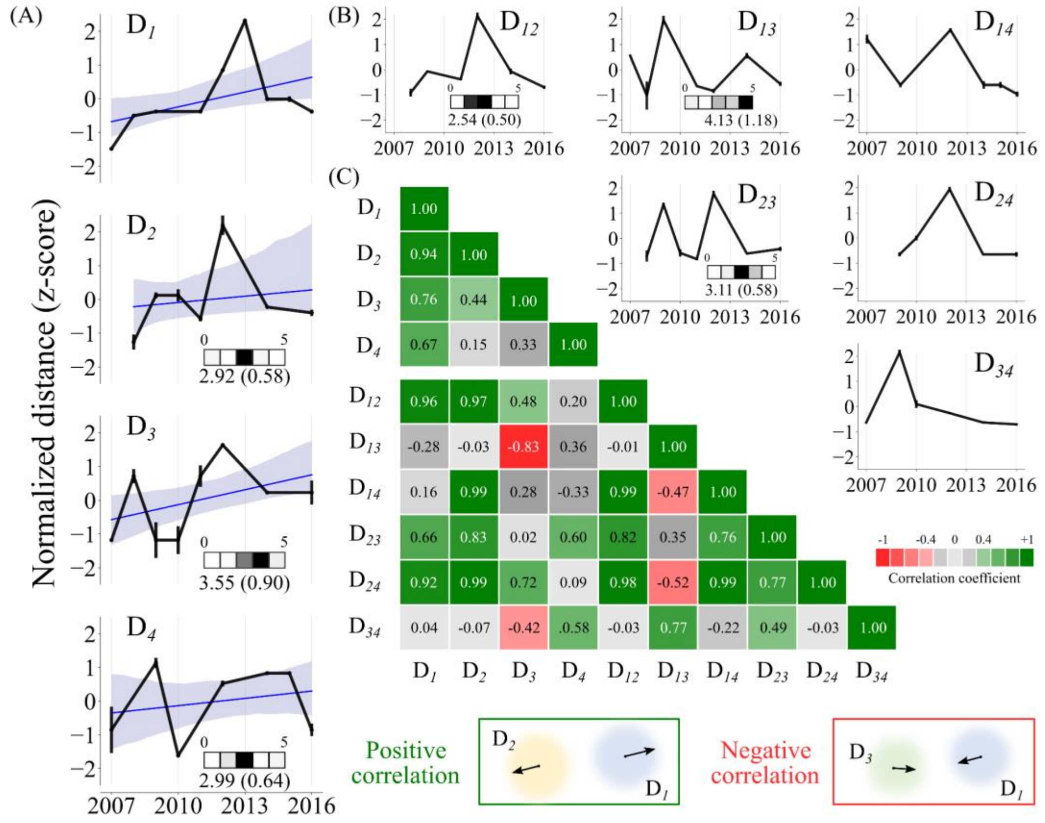

Original figure caption (Line 538-551)

Fig 4. Dynamics of E gene amino acid variation in South India between 2007 and 2016. (A) Normalized amino acid distances from the strains isolated in 2007/2008. Hamming distances were normalized with the sequence length and converted to a z-score. Distances were bootstrap sampled (n = 100) to calculate the reported median. Error bars represent standard error. The blue line represents a linear regression fit to the data with 80% confidence interval(B) Year-wise normalised interserotype distances (as z-score) are reported as calculated by randomly selecting the sequences from each serotype every year (100 bootstraps). The inset heatmap depicts the time period of oscillation binned yearly (range: 0 to 5 years). The mean and standard deviation of the time period distribution is denoted next to the heatmap. (C) Distribution of correlation coefficients for within-serotype dynamics (highlighted in yellow) and inter-serotype distance dynamics. Histograms were obtained by deleting up to 3 data points randomly (1000 bootstraps). D_i indicates the distance dynamics between DENVi and its ancestral sequence. D_{ij} indicates the interserotype distance dynamics between *i*-th and *j*-th serotype. Green and red indicate positive ($\rho > 0.4$) and negative correlation ($\rho < -0.4$), respectively, while grey indicates weak or no correlation.

Revised figure caption (Line 550-563)

Fig 4. Dynamics of E gene amino acid variation in South India between 2007 and 2016.

(A) Normalized amino acid distances with respect to the 2007/2008 dengue strains is plotted in black. Hamming distances were normalized with the sequence length and converted to a z-score. Distances were bootstrap sampled (n = 100) to calculate the reported median. Error bars represent standard error. The blue line represents a linear regression fit to the data with 80% (gray shade) confidence interval. Di indicates the distance dynamics between DENVi from its (2007/2008) ancestral sequence. (B) Year-wise normalised interserotype distances (as z-score) calculated by randomly selecting the sequences from each serotype every year (100 bootstraps) is plotted. The inset heatmap depicts the time period of oscillation binned yearly (range of 0 to 5 years). The mean and standard deviation (in brackets) of the time period distribution is denoted next to the heatmap. D_{ij} indicates the interserotype distance dynamics between *i*-th and *j*-th serotype. (C) Median of the Pearson's correlation coefficients between the distance dynamics D_i and D_{ij} is shown as a heatmap with corresponding colormap. A schematic depicting the relative evolution of dengue serotypes in two-dimensional sequence space with positive and negative correlation between D_{ij} and D_i or D_j is shown at the bottom. Central point represents the ancestral strain, and the arrow represents the direction of the virus evolution.

On page 17, it was unclear to me what 'dominant' amino acids were, and what a >50% of the differences therefore actually means? What is the denominator here?

We thank the reviewer for pointing this out, we agree that the 'Dominant' word is misleading in this context and we have changed it to 'significant' amino acid differences.

Sites with significant amino acid differences mean the positions where >10% of the sequences from India show a different amino acid compared to the vaccine strain. This has been added to the methods section.

>50% refers to the percentage of the total significant differences that are on the exposed part of the E protein. We have rephrased the sentence to make it less confusing.

Methods: Original statement (Line 211-212)

Sites with differences in >10% of sequences were mapped onto the envelope protein structure using PyMOL (v2.4.1) [49].

Methods: Revised statement (Line 209-210)

Sites with differences in >10% of sequences were identified as significant differences and were mapped onto the envelope protein structure using PyMOL (v2.4.1) [54].

Results: Original statement (Line 413-416)

Mapping of dominant amino acid differences (present in >10% sequences) on the envelope structures shows that ≥50% of the differences (50% DENV1, 68% DENV2, 92% DENV3 and 50% DENV4) lie on the exposed surface of the envelope protein on the virus, which is accessible to the majority of the antibodies (Fig 5B, S11 Fig, S6 Table).

Results: Revised statement (Line 416-418)

More than half of the significant amino acid differences (50% DENV1, 68% DENV2, 92% DENV3 and 50% DENV4) lie on the exposed surface of the envelope protein on the virus, which is accessible to the majority of the antibodies (Fig 5B, S12 Fig, S7 Table).

Again just because they are genetically different from vaccine strains, doesn't necessarily mean there will be reduced efficacy.

We agree with the reviewer that different strains need not have drastically different vaccine efficacies. However, we provide several references where vaccine efficacy for mutations observed suggest reduction in efficacy. We have discussed this part earlier and reproducing below for the convenience of the reviewer.

We show that out of 12 strains from 3 commercial vaccines that we considered, only 1 strain represents the genotype circulating in India over the last 20 years. Such antibody neutralization and vaccine efficacy studies for Indian viruses have not been conducted till date. Therefore, we have relied on comparison of vaccine efficacy from other countries against different strains to draw our conclusions.

The genotype differences can cause significant drop in antibody titers and vaccine efficacies (Bell et al., 2019; Juraska et al., 2018; Martinez et al., 2020). We have discussed this aspect in our manuscript especially in the context of the mutations observed in Indian dengue variants (Line 432-443). Briefly, previous studies have shown up to two-log reduction in neutralization antibody titers with different genotypes (D. et al., 2010; Martinez et al., 2020). Similarly, human and macaque challenge studies (Kirkpatrick et al., 2016; Osorio et al., 2011) showed complete protection against the infection against the vaccine genotype while only partial protection against infection with a different genotype. We have also discussed a mutational sieve analysis for DENV4 vaccine (Juraska et al., 2018) which shows reduced vaccine efficacy caused due to differences in genotypes.

Results: Revised statement (Line 431-434)

Therefore, antigenic differences of Indian dengue viruses with respect to the vaccines define the majority of E protein differences. This can have important implications for vaccine efficacy, where vaccines will likely evoke poor neutralization against dengue virus in India.

In the final paragraph of the discussion, the authors conclude that their analysis shows that the immunity developed by the vaccine can also impact the future course of dengue evolution. While this may be true, I could not see how their analysis has shown this.

As we have seen in the previous results, the viruses evolve to become different or similar to its ancestors or other serotypes. The vaccination will induce antibodies which can neutralize the vaccine strain more efficiently and cross reactive antibodies against other genotypes at lower levels. We believe this can have an impact on the course of subsequent dengue evolution in complex ways. Nevertheless, we agree that our data analysis does not directly show this and we have revised the statement.

Discussion: Original statement (Line 503-505)

One can argue that regionally tailored vaccines would be more efficacious due to the geographical restriction of dengue [77]. However, our evolutionary analysis suggests that the immunity developed by vaccination can also impact the future course of dengue virus evolution.

Discussion: Revised statement (Line 511-513)

One can argue that regionally tailored vaccines would be more efficacious due to the geographical restriction of dengue [83]. However, the impact of immunity developed by vaccination on the future course of dengue virus evolution needs to be considered carefully.

References

Alagarasu, K., Patil, J. A., Kakade, M. B., More, A. M., Yogesh, B., Newase, P., Jadhav, S. M., Parashar, D., Kaur, H., Gupta, N., Vijay, N., Narayan, J., Shah, P. S., Sharma, D. A., Jain, D. A., Bhargava, D. A., Anukumar, D. B., M, D. A., Malhotra, D. B., … Vijayachari, D. P. (2021). Serotype and genotype diversity of dengue viruses circulating in India: a multi-centre retrospective study involving the Virus Research Diagnostic Laboratory Network in 2018. *International Journal of Infectious Diseases*, *111*, 242–252. https://doi.org/10.1016/j.ijid.2021.08.045

Bell, S. M., Katzelnick, L., & Bedford, T. (2019). Dengue genetic divergence generates within-serotype antigenic variation, but serotypes dominate evolutionary dynamics. *ELife*, *8*, e42496. https://doi.org/10.7554/eLife.42496

Cai, Y., Zhang, J., Xiao, T., Lavine, C. L., Rawson, S., Peng, H., Zhu, H., Anand, K., Tong, P., Gautam, A., Lu, S., Sterling, S. M., Walsh, R. M., Rits-Volloch, S., Lu, J., Wesemann, D. R., Yang, W., Seaman, M. S., & Chen, B. (2021). Structural basis for enhanced infectivity and immune evasion of SARS-CoV-2 variants. In *bioRxiv : the preprint server for biology*. https://doi.org/10.1101/2021.04.13.439709

D., B. J., Kyle, A. S., Soila, S.-P., M., O. K., Syd, J., H., F. D., & S., D. M. (2010). Genotype-specific neutralization and protection by antibodies against dengue virus type 3. *Journal of Virology*, *84*(20), 10630–10643. https://doi.org/10.1128/JVI.01190-10

Duong, V., Lambrechts, L., Paul, R. E., Ly, S., Lay, R. S., Long, K. C., Huy, R., Tarantola, A., Scott, T. W., Sakuntabhai, A., & Buchy, P. (2015). Asymptomatic humans transmit dengue virus to mosquitoes. *Proceedings of the National Academy of Sciences*, *112*(47), 14688–14693. https://doi.org/10.1073/pnas.1508114112

Goncalvez, A. P., Engle, R. E., St. Claire, M., Purcell, R. H., & Lai, C.-J. (2007). Monoclonal antibody-mediated enhancement of dengue virus infection in vitro and in vivo and strategies for prevention. *Proceedings of the National Academy of Sciences*, *104*(22), 9422–9427. https://doi.org/10.1073/pnas.0703498104

Halstead, S. B. (1979). In Vivo Enhancement of Dengue Virus Infection in Rhesus Monkeys by Passively Transferred Antibody. *The Journal of Infectious Diseases*, *140*(4), 527–533. https://doi.org/10.1093/infdis/140.4.527

Huang, A. T., Salje, H., Escoto, A. C., Chowdhury, N., Chávez, C., Garcia-Carreras, B., Rutvisuttinunt, W., Maljkovic Berry, I., Gromowski, G. D., Wang, L., Klungthong, C., Thaisomboonsuk, B., Nisalak, A., Trimmer-Smith, L. M., Rodriguez-Barraquer, I., Ellison, D. W., Jones, A. R., Fernandez, S., Thomas, S. J., … Katzelnick, L. C. (2022). Beneath the

surface: Amino acid variation underlying two decades of dengue virus antigenic dynamics in Bangkok, Thailand. *PLOS Pathogens*, *18*(5), e1010500. https://doi.org/10.1371/journal.ppat.1010500

Juraska, M., Magaret, C. A., Shao, J., Carpp, L. N., Fiore-Gartland, A. J., Benkeser, D., Girerd-Chambaz, Y., Langevin, E., Frago, C., Guy, B., Jackson, N., Duong Thi Hue, K., Simmons, C. P., Edlefsen, P. T., & Gilbert, P. B. (2018). Viral genetic diversity and protective efficacy of a tetravalent dengue vaccine in two phase 3 trials. *Proceedings of the National Academy of Sciences*, *115*(36), E8378–E8387. https://doi.org/10.1073/pnas.1714250115

Katzelnick, L. C., Fonville, J. M., Gromowski, G. D., Arriaga, J. B., Green, A., James, S. L., Lau, L., Montoya, M., Wang, C., VanBlargan, L. A., Russell, C. A., Thu, H. M., Pierson, T. C., Buchy, P., Aaskov, J. G., Muñoz-Jordán, J. L., Vasilakis, N., Gibbons, R. V, Tesh, R. B., … Smith, D. J. (2015). Dengue viruses cluster antigenically but not as discrete serotypes. *Science*, *349*(6254), 1338–1343. https://doi.org/10.1126/science.aac5017

Kirkpatrick, B. D., Whitehead, S. S., Pierce, K. K., Tibery, C. M., Grier, P. L., Hynes, N. A., Larsson, C. J., Sabundayo, B. P., Talaat, K. R., Janiak, A., Carmolli, M. P., Luke, C. J., Diehl, S. A., & Durbin, A. P. (2016). The live attenuated dengue vaccine TV003 elicits complete protection against dengue in a human challenge model. *Science Translational Medicine*, *8*(330), 330ra36-330ra36. https://doi.org/10.1126/scitranslmed.aaf1517

Lin, Y.-W., Wang, K.-J., Lei, H.-Y., Lin, Y.-S., Yeh, T.-M., Liu, H.-S., Liu, C.-C., & Chen, S.-H. (2002). Virus replication and cytokine production in dengue virus-infected human B lymphocytes. *Journal of Virology*, *76*(23), 12242–12249. https://doi.org/10.1128/jvi.76.23.12242-12249.2002

Martinez, D. R., Yount, B., Nivarthi, U., Munt, J. E., Delacruz, M. J., Whitehead, S. S., Durbin, A. P., de Silva, A. M., & Baric, R. S. (2020). Antigenic variation of the dengue virus 2 genotypes impacts the neutralization activity of human antibodies in vaccinees. *Cell Reports*, *33*(1), 108226. https://doi.org/10.1016/j.celrep.2020.108226

Murhekar, M. V., Kamaraj, P., Kumar, M. S., Khan, S. A., Allam, R. R., Barde, P., Dwibedi, B., Kanungo, S., Mohan, U., Mohanty, S. S., Roy, S., Sagar, V., Savargaonkar, D., Tandale, B. V., Topno, R. K., Sapkal, G., Kumar, C. P. G., Sabarinathan, R., Kumar, V. S., … Mehendale, S. M. (2019). Burden of dengue infection in India, 2017: a cross-sectional population based serosurvey. *The Lancet Global Health*, *7*(8). https://doi.org/10.1016/S2214-109X(19)30250-5

Nguyet, M. N., Duong, T. H. K., Trung, V. T., Nguyen, T. H. Q., Tran, C. N. B., Long, V. T., Dui, L. T., Nguyen, H. L., Farrar, J. J., Holmes, E. C., Rabaa, M. A., Bryant, J. E., Nguyen, T. T., Nguyen, H. T. C., Nguyen, L. T. H., Pham, M. P., Nguyen, H. T., Luong, T. T. H., Wills, B., … Simmons, C. P. (2013). Host and viral features of human dengue cases shape the population of infected and infectious Aedes aegypti mosquitoes. *Proceedings of the National Academy of Sciences of the United States of America*, *110*(22), 9072–9077. https://doi.org/10.1073/pnas.1303395110

Osorio, J. E., Brewoo, J. N., Silengo, S. J., Arguello, J., Moldovan, I. R., Tary-Lehmann, M., Powell, T. D., Livengood, J. A., Kinney, R. M., Huang, C. Y.-H., & Stinchcomb, D. T. (2011). Efficacy of a tetravalent chimeric dengue vaccine (DENVax) in Cynomolgus Macaques. *The American Society of Tropical Medicine and Hygiene*, *84*(6), 978–987. https://doi.org/10.4269/ajtmh.2011.10-0592

Pybus, O. G., & Rambaut, A. (2009). Evolutionary analysis of the dynamics of viral infectious disease. *Nature Reviews Genetics*, *10*(8), 540–550. https://doi.org/10.1038/nrg2583

Quinn, M., Kou, Z., Martinez-Sobrido, L., Schlesinger, J. J., & Jin, X. (2013). Increased Virus Uptake Alone is Insufficient to Account for Viral Burst Size Increase during Antibody-Dependent Enhancement of Dengue Viral Infection. *Journal of Immunological Techniques & Infectious Diseases*, *2*(3). https://doi.org/10.4172/2329-9541.1000111

Rubio-Casillas, A., Redwan, E. M., & Uversky, V. N. (2022). SARS-CoV-2: A Master of Immune Evasion. *Biomedicines*, *10*(6). https://doi.org/10.3390/biomedicines10061339

Sang, S., Liu-Helmersson, J., Quam, M. B. M., Zhou, H., Guo, X., Wu, H., & Liu, Q. (2019). The evolutionary dynamics of DENV 4 genotype I over a 60-year period. *PLoS Neglected Tropical Diseases*, *13*(7). https://doi.org/10.1371/journal.pntd.0007592

Yamanaka, A., Imad, H. A., Phumratanaprapin, W., Phadungsombat, J., Konishi, E., & Shioda, T. (2021). Antibody-dependent enhancement representing in vitro infective progeny virus titer correlates with the viremia level in dengue patients. *Scientific Reports*, *11*(1), 12354. https://doi.org/10.1038/s41598-021-91793-0
